# PGD_2_ and CRTH2 counteract Type 2 cytokine–elicited intestinal epithelial responses during helminth infection

**DOI:** 10.1084/jem.20202178

**Published:** 2021-07-20

**Authors:** Oyebola O. Oyesola, Michael T. Shanahan, Matt Kanke, Bridget M. Mooney, Lauren M. Webb, Shuchi Smita, Macy K. Matheson, Pamela Campioli, Duc Pham, Simon P. Früh, John W. McGinty, Madeline J. Churchill, Jordan L. Cahoon, Pavithra Sundaravaradan, Becca A. Flitter, Karthik Mouli, Marija S. Nadjsombati, Elena Kamynina, Seth A. Peng, Rebecca L. Cubitt, Karsten Gronert, James D. Lord, Isabella Rauch, Jakob von Moltke, Praveen Sethupathy, Elia D. Tait Wojno

**Affiliations:** 1 Department of Immunology, University of Washington, Seattle, WA; 2 Baker Institute for Animal Health and Department of Microbiology and Immunology, Cornell University College of Veterinary Medicine, Ithaca, NY; 3 Department of Biomedical Sciences, Cornell University College of Veterinary Medicine, Ithaca, NY; 4 Department of Molecular Microbiology and Immunology, Oregon Health and Science University, Portland, OR; 5 Vision Science Program, School of Optometry, University of California, Berkeley, Berkeley, CA; 6 Benaroya Research Institute at Virginia Mason Medical Center, Division of Gastroenterology, Seattle, WA

## Abstract

Type 2 inflammation is associated with epithelial cell responses, including goblet cell hyperplasia, that promote worm expulsion during intestinal helminth infection. How these epithelial responses are regulated remains incompletely understood. Here, we show that mice deficient in the prostaglandin D_2_ (PGD_2_) receptor CRTH2 and mice with CRTH2 deficiency only in nonhematopoietic cells exhibited enhanced worm clearance and intestinal goblet cell hyperplasia following infection with the helminth *Nippostrongylus brasiliensis*. Small intestinal stem, goblet, and tuft cells expressed CRTH2. CRTH2-deficient small intestinal organoids showed enhanced budding and terminal differentiation to the goblet cell lineage. During helminth infection or in organoids, PGD_2_ and CRTH2 down-regulated intestinal epithelial *Il13ra1* expression and reversed Type 2 cytokine–mediated suppression of epithelial cell proliferation and promotion of goblet cell accumulation. These data show that the PGD_2_–CRTH2 pathway negatively regulates the Type 2 cytokine–driven epithelial program, revealing a mechanism that can temper the highly inflammatory effects of the anti-helminth response.

## Teaser

Type 2 cytokines promote epithelial changes that help to expel worms during intestinal helminth infection. Oyesola et al. show that prostaglandin D_2_ and its receptor CRTH2 are novel negative regulators of this response, dampening the Type 2 cytokine–mediated program.

## Introduction

Type 2 inflammation occurs in response to intestinal helminth infection and is critical for parasite expulsion and tissue repair. Helminth products and infection-induced intestinal damage cause release of the cytokines IL-25, IL-33, and thymic stromal lymphopoietin from epithelial and other cells. These factors drive activation of immune cells such as group 2 innate lymphoid cells (ILC2s) and CD4^+^ T helper type 2 (Th2) cells that secrete the Type 2 cytokines IL-4, IL-5, IL-9, and IL-13 (Allen and Maizels, 2011; Maizels et al., 2012; Paul and Zhu, 2010; Pulendran and Artis, 2012; Stetson et al., 2004). Helminth infection and associated IL-13 responses (Bancroft et al., 1998; McKenzie et al., 1998) trigger changes in the intestinal epithelium, including increased epithelial cell proliferation and movement through the villus in the epithelial escalator (Cliffe et al., 2005), increased intestinal permeability (Zhao et al., 2003), crypt hyperplasia and villus atrophy (Artis and Grencis, 2008; McDermott et al., 2005), tuft cell responses (Gerbe et al., 2016; Howitt et al., 2016; von Moltke et al., 2016), and goblet cell hyperplasia and mucin responses (Hasnain et al., 2012), all of which are important in the “weep and sweep” response that leads to effective worm expulsion (Allen and Maizels, 2011; Coakley and Harris, 2020; Oyesola et al., 2020b; Pulendran and Artis, 2012). Many of these effects are due to IL-13R signaling on intestinal stem cells (ISCs), with IL-13 stimulation causing ISCs to skew toward goblet (Hasnain et al., 2012; McKenzie et al., 1998) and tuft (Biton et al., 2018; Gerbe et al., 2016; Howitt et al., 2016; von Moltke et al., 2016) cell differentiation in vivo and depleting the renewal capacity of the Lgr5^+^ stem cell pool in vitro (Biton et al., 2018).

Type 2 inflammation–induced changes in the intestinal epithelium must be carefully regulated to prevent inappropriate inflammation and maintain a functional epithelium and ISC pool (Allen and Maizels, 2011; Coakley and Harris, 2020; Oyesola et al., 2020b; Pulendran and Artis, 2012; Sorobetea et al., 2018). Inhibitory cytokines such as IL-10 and TGF-β, the activities of T regulatory cells, Type 1 cytokine antagonism of IL-4 function, expression of the IL-13 decoy receptor (IL-13Rα2), and RELMα from alternatively activated macrophages can all temper the Type 2 milieu that acts on intestinal epithelial cells (IECs; Gieseck et al., 2018). In addition, a recent study has shown that IL-13 signaling activates BMP2 in IECs to limit IL-13–mediated tuft cell accumulation in a negative feedback loop (Lindholm et al., 2020* Preprint*). However, our understanding of the factors that act directly on IECs to counteract the changes provoked by Type 2 cytokines is incomplete.

Eicosanoids are bioactive lipids derived from arachidonic acids that have both pro- and anti-inflammatory functions. These factors are highly labile yet accumulate to high concentrations in tissue microenvironments, making them well suited to regulate immune and epithelial responses during inflammation (Harris et al., 2002; Serhan et al., 2015). Prostaglandins are eicosanoids synthesized by cyclooxygenase enzymes and specific prostaglandin synthases (Harris et al., 2002; Serhan et al., 2015), with elevated concentrations of prostaglandin D_2_ (PGD_2_) associated with Type 2 inflammation (Balzar et al., 2011; Fajt et al., 2013; Konya and Mjösberg, 2016). Immune cells including mast cells may be a source of PGD_2_ (Lewis et al., 1982; MacDermot et al., 1984; Tanaka et al., 2000), but epithelial cells, specifically tuft cells, may also be key sources (Bezençon et al., 2008; DelGiorno et al., 2020; Gerbe et al., 2016; Haber et al., 2017).

Previous studies have shown that PGD_2_ ligates one of its two receptors, the chemoattractant receptor-homologous molecule expressed on Th2 cells (CRTH2), to induce migration and production of Type 2 cytokines in eosinophils, ILC2s, basophils, and Th2 cells (Hirai et al., 2001; Nagata et al., 1999; Oyesola et al., 2020b; Pettipher, 2008), particularly in the lung (Oyesola et al., 2020a; Tait Wojno et al., 2015). Based on these findings, CRTH2 inhibitors have been used to treat humans with allergic airway disease (Barnes et al., 2012; Marone et al., 2019). These data define a pro-inflammatory role for the PGD_2_–CRTH2 pathway in the lung, acting on immune cells. However, other prostaglandin family members and PGD_2_ itself, acting through its other receptor DP1, can also play pro-resolving or anti-inflammatory roles in the steady state and during inflammation (de los Reyes Jiménez et al., 2020; Iwanaga et al., 2014; Murata et al., 2013; Nakamura et al., 2015; Yamamoto et al., 2011). The predicted capacity for intestinal tuft cells to produce PGD_2_ (Bezençon et al., 2008; DelGiorno et al., 2020; Gerbe et al., 2016; Haber et al., 2017) suggests a potential role for the PGD_2_–CRTH2 pathway in the intestine during helminth infection. However, the function of PGD_2_ and CRTH2 in the intestine is unclear.

Here, we define a previously unrecognized role for the PGD_2_–CRTH2 pathway in acting on IECs to limit the Type 2 cytokine–induced functional program. Unexpectedly, despite the known pro-inflammatory role for CRTH2 (Oyesola et al., 2020a; Tait Wojno et al., 2015), mice deficient in CRTH2 and mice that lacked CRTH2 only in the radioresistant compartment had enhanced worm expulsion, largely intact Type 2 cytokine responses, and increased goblet cell hyperplasia compared with WT control mice following infection with the helminth *Nippostrongylus brasiliensis*. ISCs, goblet cells, and tuft cells expressed *Gpr44*, the gene that encodes CRTH2. CRTH2-deficient compared with WT small intestinal organoids exhibited enhanced budding and goblet cell frequencies. In vivo, WT mice that have an operational PGD_2_–CRTH2 pathway had elevated IEC proliferation following infection, while *Gpr44^−/−^* mice that have similar levels of Type 2 cytokines but lack signals from PGD_2_ via CRTH2 had less proliferation. Similarly, in vitro in small intestinal organoids, the PGD_2_–CRTH2 pathway reversed Type 2 cytokine–mediated decreases in IEC proliferation and increases in goblet cell accumulation and decreased expression of *Il13ra1*, counteracting specific aspects of the Type 2 cytokine–elicited response. Taken together, these findings reveal a previously unappreciated role for the PGD_2_–CRTH2 pathway in directing IEC responses during Type 2 inflammation and define a new mechanism that negatively regulates the Type 2 cytokine–elicited IEC program.

## Results

### CRTH2 deficiency results in enhanced *N. brasiliensis* expulsion

**Figure 1.**
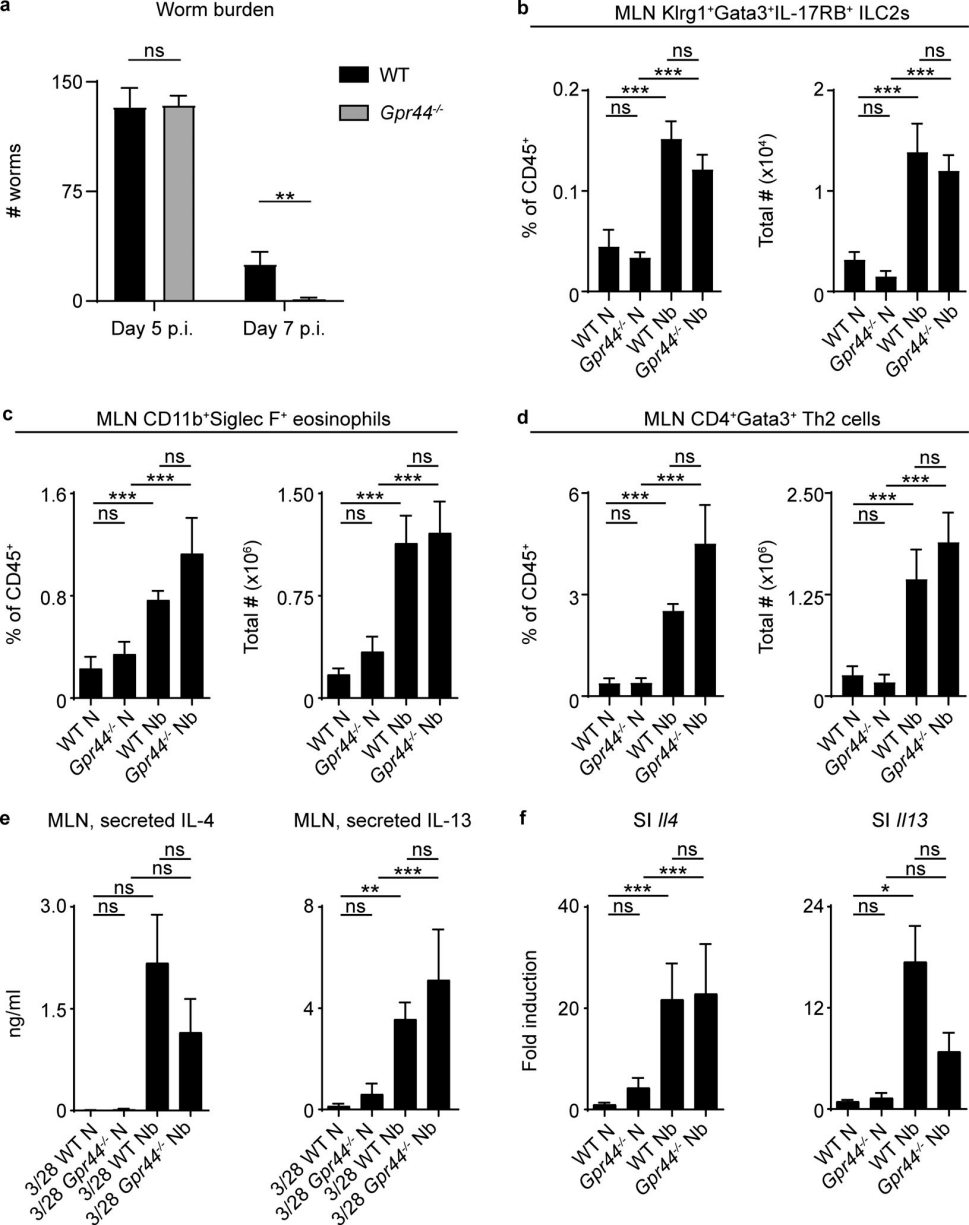
**CRTH2 deficiency results in enhanced worm expulsion from the small intestine.(a)** Littermate or cohoused C57BL/6 WT and *Gpr44^−/−^* mice were infected with 500 *N. brasiliensis* (Nb) L3 larvae subcutaneously, and on day 5 and day 7 p.i., intestinal worm burden was quantified. **(b–d)** On day 7 p.i., the percentage and total number of ILC2s (live, CD45^+^Lin^−^Klrg1^+^IL-17RB^+^Gata3^+^; b), eosinophils (live, CD45^+^Siglec F^+^CD11b^+^; c), and CD4^+^ Th2 cells (live, CD45^+^Lin^+^CD4^+^Gata3^+^; d) in the MLNs were assessed using flow cytometry. **(e and f)** On day 7 p.i., levels of IL-4 and IL-13 in the cell-free supernatant of MLN cells stimulated for 48 h with anti-CD3/anti-CD28 (3/28) were measured by ELISA (e), and the expression of *Il4* and *Il13* in small intestinal (SI) homogenates was measured by real-time PCR (relative to *Actβ* and normalized to WT naive [N]; f). Data are mean ± SEM. **(a–f)** Analyzed using a linear mixed-effects model with pairwise comparison. **(a)** Day 5 p.i., *n* = 4–6; two independent experiments. Day 7 p.i., *n* = 10 or 11; four independent experiments. **(b)** N, *n* = 3 or 4; Nb, *n* = 5 or 6; two independent experiments. **(c)** N, *n* = 7–9; Nb, *n* = 12–14; six independent experiments. **(d)** N, *n* = 5 or 6; Nb, *n* = 8–12; three independent experiments. **(e)** IL-4, N, *n* = 7; Nb, *n* = 8–11; four independent experiments. IL-13, N, *n* = 5 or 6; Nb, *n* = 6–8; three independent experiments. **(f)** *Il4* N, *n* = 5–7; Nb, *n* = 7; three independent experiments. *Il13* N, *n* = 6; Nb, *n* = 10–13; four independent experiments. Each *n* refers to number of mice/group in total across all experiments. *, P ≤ 0.05; **, P ≤ 0.01; ***, P ≤ 0.001.

**Figure S1.**
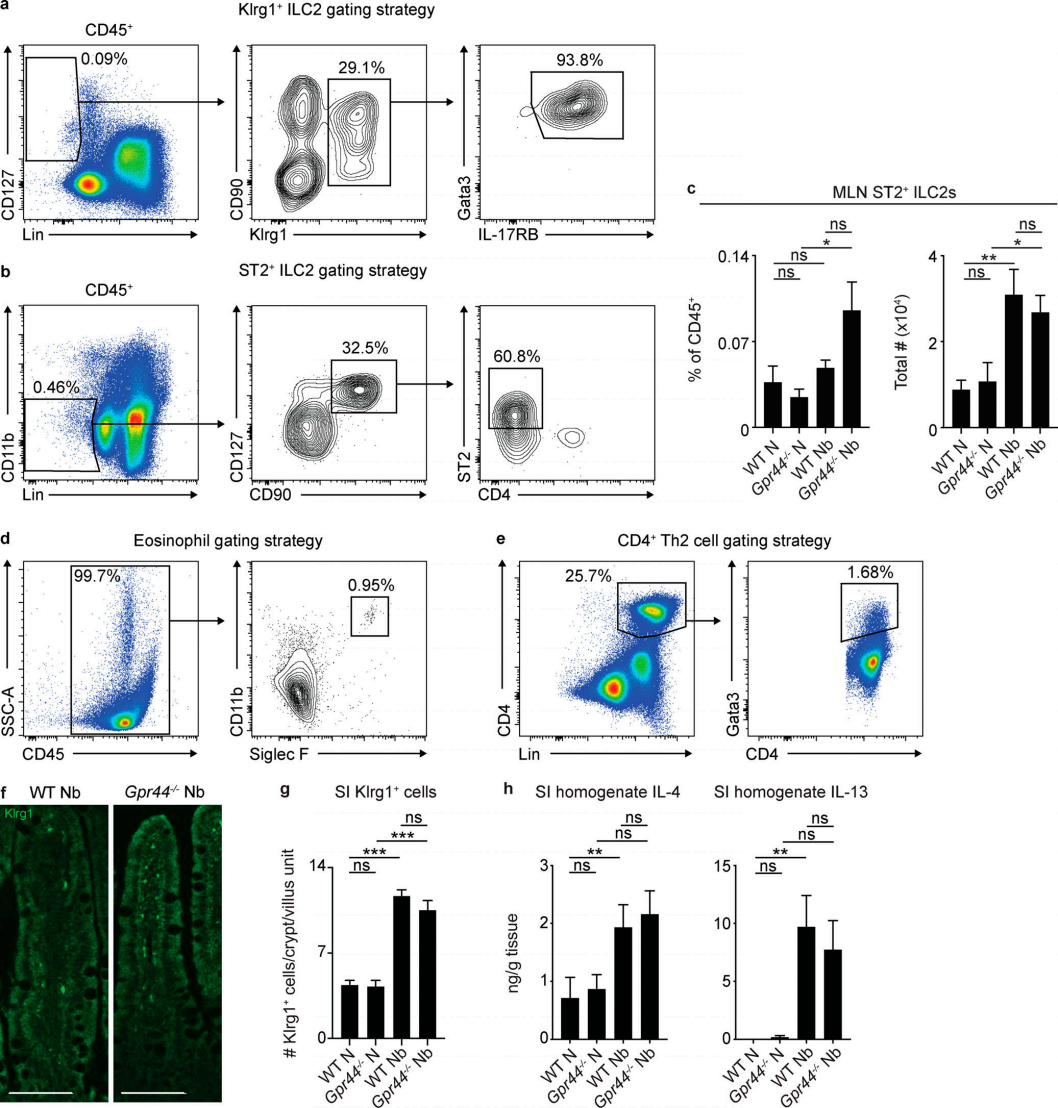
**ILC2, eosinophil, Th2 cell, and small intestinal Type 2 cytokine responses in CRTH2-deficient mice.** C57BL/6 WT and *Gpr44^−/−^* mice were infected with 500 *N. brasiliensis* (Nb) L3 larvae subcutaneously. **(a and b)** ILC2s in the MLN were gated as live, CD45^+^Lin(CD3/CD5/TCRγδ/CD11b/CD11c/CD19/NK1.1)*^−^*CD127^+^Klrg1^+^Gata3^+^IL-17RB^+^ (a) or live, CD45^+^Lin(CD3/CD5/CD11b/CD11c/CD19/NK1.1)***^−^***CD127^+^CD90^+^ST2^+^CD4*^−^* (b). **(c)** On day 7 p.i., the percentage and total number of ILC2s (live, CD45^+^Lin***^−^***CD127^+^CD90^+^CD25^+^ST2^+^CD4***^−^***) in the MLN were assessed using flow cytometry. **(d)** Eosinophils in MLN were gated as live, CD45^+^CD11b^+^Siglec F^+^. **(e)** CD4^+^ Th2 cells in the MLN were gated as live, CD45^+^Lin(CD3/CD5/CD11b/CD11c/CD19/NK1.1)^+^CD4^+^Gata3^+^. **(f and g)** On day 5 p.i., representative images showing immunofluorescent staining for Klrg1 in histological sections of the small intestine (SI; f) were quantified for the number of Klrg1^+^ cell per crypt/villus unit (g). Scale bar = 100 µm. **(h)** On day 7 p.i., IL-4 and IL-13 in small intestinal homogenates were measured by ELISA. Data are mean ± SEM. **(a, b, d, and e)** Representative plots. **(c, g, and h)** Analyzed using a linear mixed-effects model with pairwise comparison. **(c)** Naive (N), *n* = 7; Nb, *n* = 11–13; four independent experiments. **(f and g)** N, *n* = 5 or 6; Nb, *n* = 5 or 6 (at least 10 crypt/villus units were examined/mouse, and those values were averaged); two independent experiments. **(h)** IL-4 N, *n* = 5–9; Nb, *n* = 14; five independent experiments. IL-13 N, *n* = 6 or 7; Nb, *n* = 12–14; five independent experiments. Each *n* refers to number of mice/group in total across all experiments. *, P ≤ 0.05; **, P ≤ 0.01; ***, P ≤ 0.001. SSC-A, side scatter A.

To begin to investigate how the PGD_2_–CRTH2 pathway affects helminth-induced Type 2 inflammation, we infected WT and CRTH2-deficient mice with the helminth parasite *N. brasiliensis*. Despite the role for CRTH2 in promoting pulmonary Type 2 inflammation (Oyesola et al., 2020a; Tait Wojno et al., 2015), CRTH2-deficient (*Gpr44^−/−^*) mice showed enhanced worm clearance compared with WT mice at day 7 postinfection (p.i.), with similar burdens at day 5 p.i. (Fig. 1 a), suggesting a suppressive role for CRTH2 in the intestine. On day 7 p.i., *Gpr44^−/−^* and WT mice had similar frequencies and total numbers of immune cells that can express CRTH2 (Oyesola et al., 2020b) in the mesenteric lymph node (MLN), including ILC2s, eosinophils, and CD4^+^ Th2 cells (Fig. 1, b–d; and Fig. S1, a–e). On day 5 p.i., *Gpr44^−/−^* and WT mice also had comparable numbers of Klrg1^+^ cells (of which the majority are ILC2s; McGinty et al., 2020) in the small intestinal lamina propria (Fig. S1, f and g). Restimulated MLN cells from infected *Gpr44^−/−^* and WT mice secreted similar levels of IL-4 and IL-13 (Fig. 1 e). Infection-induced expression of *Il4* (Fig. 1 f) and IL-4 protein (Fig. S1 h) in the small intestine was similar in WT and *Gpr44^−/−^* mice. *Gpr44^−/−^* mice had slightly lower though not statistically significant infection-induced *Il13* expression (Fig. 1 f) and IL-13 protein levels (Fig. S1 h) in the small intestine compared with WT mice at day 7 p.i. Thus, the immune response in *Gpr44^−/−^* mice was largely intact, yet they unexpectedly demonstrated enhanced clearance of *N. brasiliensis*.

### CRTH2 deficiency is associated with elevated goblet cell accumulation in the small intestine

**Figure 2.**
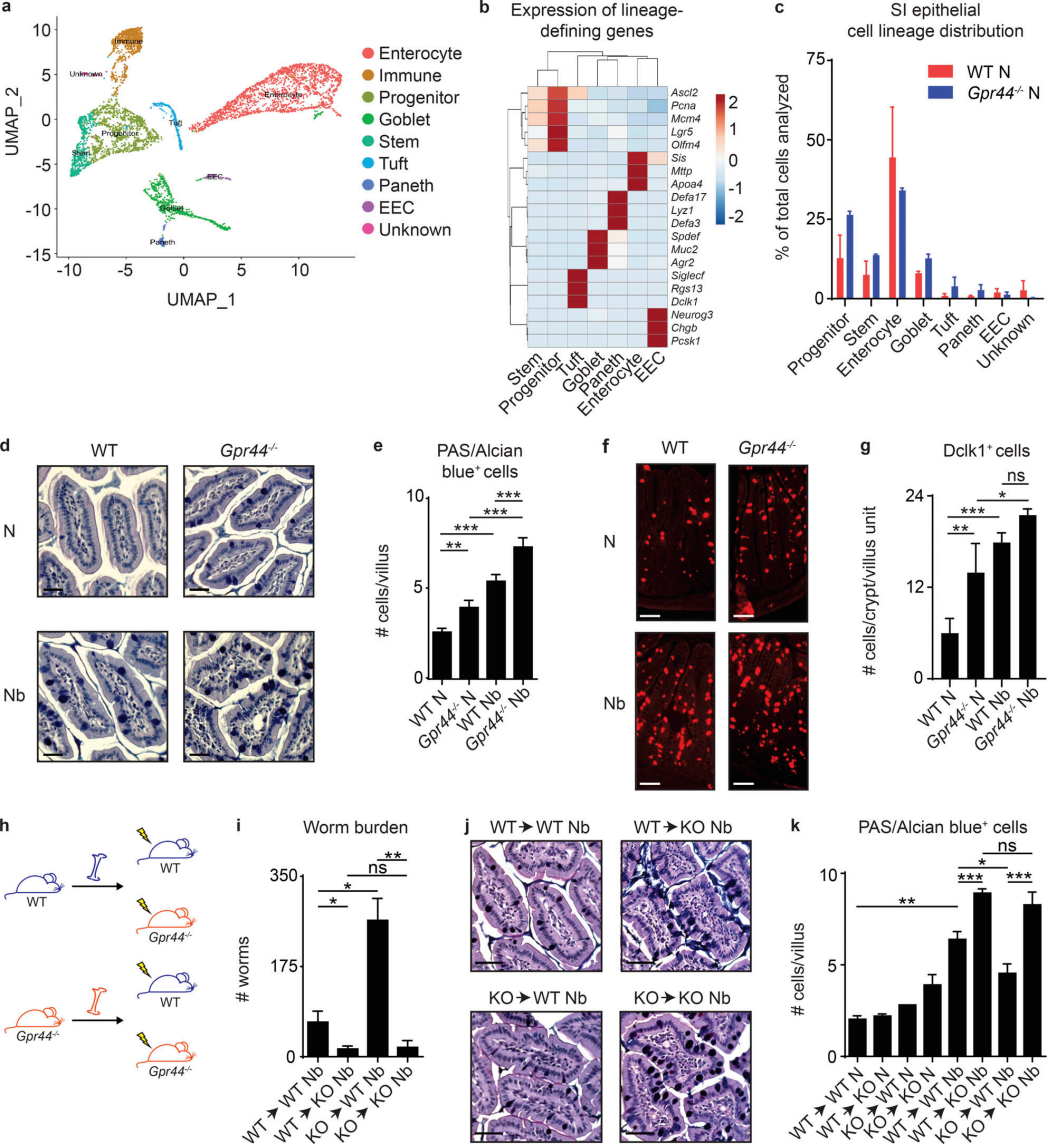
**CRTH2 deficiency in the nonhematopoietic compartment is associated with elevated secretory cell responses in the small intestine.(a–c)** Small intestinal (SI) IECs from naive (N) littermate C57BL/6 WT and *Gpr44^−/−^* mice were subjected to scRNA-seq to generate a UMAP plot of cell clusters common to all samples following cluster assignment (a; Haber et al., 2017), a heatmap of lineage-defining genes, colored by cluster assignment (b), and proportions of IEC types in WT versus *Gpr44^−/−^* mice in vivo (c). **(d–g)** Littermate or cohoused C57BL/6 WT and *Gpr44^−/−^* mice were infected with 500 *N. brasiliensis* (Nb) L3 larvae subcutaneously, and on day 7 p.i., PAS/Alcian Blue–positive cells (d and e) and Dclk1-positive cells in the small intestine (f and g) were quantified from histological sections. Scale bar = 50 µm. **(h)** Lethally irradiated littermate or cohoused WT or *Gpr44^−/−^* mice were reconstituted with littermate or cohoused WT or *Gpr44^−/−^* BM and were infected with 500 Nb L3 larvae subcutaneously. **(i–k)** On day 7 p.i., worm burden was quantified (i) and PAS/Alcian blue–positive cells in the small intestine were quantified from histological sections (j and k). Scale bar = 50 µM. Data are mean ± SEM. **(e, g, i, and k)** Analyzed using a linear mixed-effects model with pairwise comparison. **(a–c)** Two independent experiments (one mouse in each group/experiment; *n* = 4). **(d and e)** N, *n* = 3 or 4; Nb, *n* = 5 or 6; three independent experiments. **(f and g)** N, *n* = 5–7; Nb, *n* = 9; three independent experiments. **(h–k)** *n* = 2–4, two to four independent experiments. Each *n* refers to number of mice/group in total across all experiments unless otherwise noted. *, P ≤ 0.05; **, P ≤ 0.01; ***, P ≤ 0.001. EEC, enteroendocrine cell.

The above findings suggest that CRTH2 may have effects on nonimmune cell types that dictate the outcome of helminth infection, such as IECs. Consistent with this idea, single-cell RNA sequencing (scRNA-seq) analysis to identify and quantify all the major small intestinal epithelial lineages (Haber et al., 2017; Fig. 2, a and b) revealed that *Gpr44^−/−^* compared with WT mice had an increase in the percentage of ISC, goblet, tuft, and Paneth cell lineages, at the expense of enterocytes (Fig. 2 c). Due to the challenges associated with performing scRNA-seq on IECs isolated from *N. brasiliensis*–infected mice, we used histology and microscopy to assess the accumulation of goblet and tuft cells in the small intestine of *N. brasiliensis*–infected WT and *Gpr44^−/−^* mice. Periodic acid-Schiff (PAS)/Alcian blue staining revealed increased numbers of goblet cells in *Gpr44^−/−^* mice compared with WT mice in the steady state and following *N. brasiliensis* infection (Fig. 2, d and e). Immunofluorescent staining for the tuft cell marker Dclk1 revealed increased numbers of tuft cells in *Gpr44^−/−^* compared with WT mice, though only in the naive state, with no significant change in the infection-induced increase compared with WT mice (Fig. 2, f and g). These data suggest that the PGD_2_–CRTH2 pathway limits the accumulation of goblet and, to a lesser degree, tuft cells, cell types critical for worm expulsion (Gerbe et al., 2016; Hasnain et al., 2012; Howitt et al., 2016; von Moltke et al., 2016).

### CRTH2 deficiency isolated to nonhematopoietic cells results in enhanced goblet cell accumulation and *N. brasiliensis* clearance

**Figure S2.**
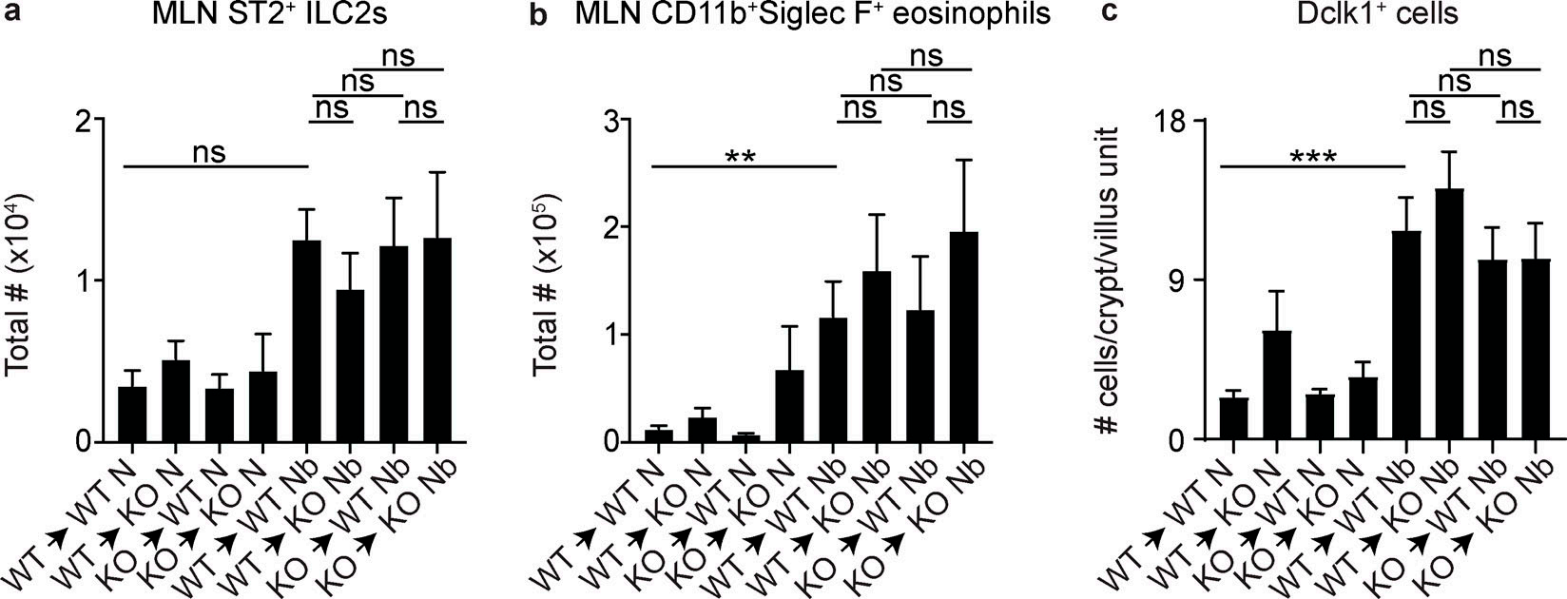
**ILC2, eosinophil, and tuft cell responses in chimeric mice.** Lethally irradiated WT or *Gpr44^−/−^* mice were reconstituted with WT or Gpr44*^−/−^* BM and were infected with 500 *N. brasiliensis* (Nb) L3 larvae subcutaneously. **(a–c)** On day 7 p.i., the total number of ILC2s (live, CD45^+^Lin*^−^*CD127^+^CD90^+^CD25^+^ST2^+^CD4*^−^*; a) and eosinophils (live, CD45^+^Siglec F^+^CD11b^+^; b) in the MLN were assessed using flow cytometry, and Dclk1-positive cells in the small intestine were quantified from histological sections (c). Data are mean ± SEM; analyzed using a linear mixed-effects model with pairwise comparison. **(a and b)** Naive (N), *n* = 5–10; Nb, *n* = 5–14; five independent experiments. **(c)** N, *n* = 5–7; Nb, *n* = 7–11; four independent experiments. Each *n* refers to number of mice/group in total across all experiments unless otherwise noted. **, P ≤ 0.01; ***, P ≤ 0.001.

To determine if enhanced worm clearance and goblet cell accumulation in *Gpr44^−/−^* mice was due to effects of CRTH2 on IECs, we first generated bone marrow (BM) chimeric mice that isolated CRTH2 deficiency to the hematopoietic or nonhematopoietic compartment (Fig. 2 h) and infected them with *N. brasiliensis*. Consistent with a role of the PGD_2_–CRTH2 pathway in the regulation of IEC responses, chimeric mice with CRTH2 deficiency only in the nonhematopoietic compartment, irrespective of the genotype of hematopoietic cells, expelled their worms more efficiently than fully WT mice (Fig. 2 i). Intriguingly, a *Gpr44^−/−^* hematopoietic system in mice with a WT nonhematopoietic compartment conferred increased susceptibility to infection (Fig. 2 i), despite no significant differences in the accumulation of ILC2s (Fig. S2 a) or eosinophils (Fig. S2 b) in the MLN between any of the groups. CRTH2 deficiency in nonhematopoietic cells alone was associated with increased numbers of PAS/Alcian blue–positive goblet cells in the small intestine compared with fully WT mice (Fig. 2, j and k). Tuft cell accumulation did not differ significantly between any of the groups in response to infection (Fig. S2 c). These results support the conclusion that CRTH2 partially suppresses nonhematopoietic IEC responses, particularly goblet cell accumulation, in the intestine during *N. brasiliensis* infection.

### Type 2 cytokines and IFN-γ regulate IEC expression of *Gpr44*

**Figure 3.**
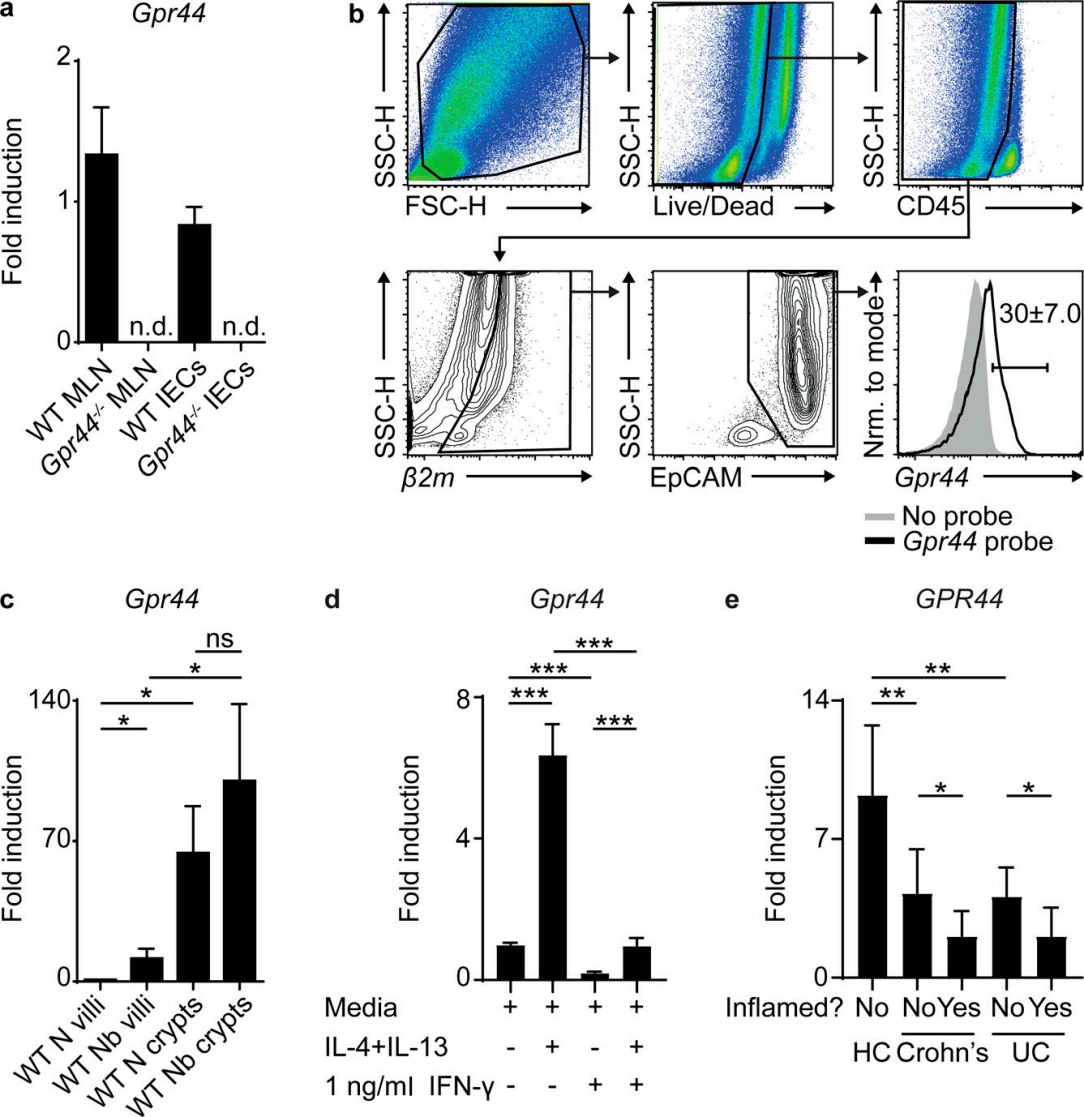
**IECs express *Gpr44* regulated by Type 1 and 2 cytokines.(a)** Littermate C57BL/6 WT and *Gpr44^−/−^* small intestinal IECs were analyzed for relative expression of *Gpr44* by real-time PCR (MLN cells as positive control, calculated relative to *Actβ*). **(b)** Gating strategy for single-cell RNA transcript staining for *Gpr44* on small intestinal IECs from WT mice that were CD45^−^, *β2M^+^,* and EpCAM^+^. Gray shaded, no probe control; black line, fully stained. *β2M^+^* cells were selected as a control for successful permeabilization and transcript labeling. **(c)** C57BL/6 WT mice were infected with 500 *N. brasiliensis* (Nb) L3 larvae subcutaneously. Small intestinal IECs from naive (N) and *N. brasiliensis–*infected mice were assessed for expression of *Gpr44* in villi and crypt fractions using real-time PCR. **(d)** Small intestinal organoids from WT mice were cultured for 2 d in media, Type 2 cytokines, and/or IFN-γ, and *Gpr44* expression was assessed. **(e)** Full transcriptome profile of *GPR44* expression was assessed from unfractionated ascending colon mucosal biopsies from healthy controls (HC) or Crohn’s disease (Crohn’s) or ulcerative colitis (UC) patient samples (affected or unaffected areas). Mouse data are mean ± SEM. **(c and d)** Analyzed using a linear mixed-effects model with pairwise comparison. Human data are mean ± SD. **(a)** *n* = 7 or 8; three independent experiments. **(b)** Representative plots, *n* = 6; three independent experiments. **(c)** *n* = 4 or 5; five independent experiments. **(d)** Two different data points/group from pooled organoids from each experiment, two or three independent experiments. Each *n* refers to number of mice/group in total across all experiments unless otherwise noted. **(e)** HC, *n* = 13; Crohn’s (9 inflamed, 7 uninflamed), *n* = 16; and UC (10 inflamed, 8 uninflamed), *n* = 18. *, P ≤ 0.05; **, P ≤ 0.01; ***, P ≤ 0.001. FSC-H, forward scatter H; Nrm., normalized; n.d., not detected; SSC-H, side scatter H.

**Figure S3.**
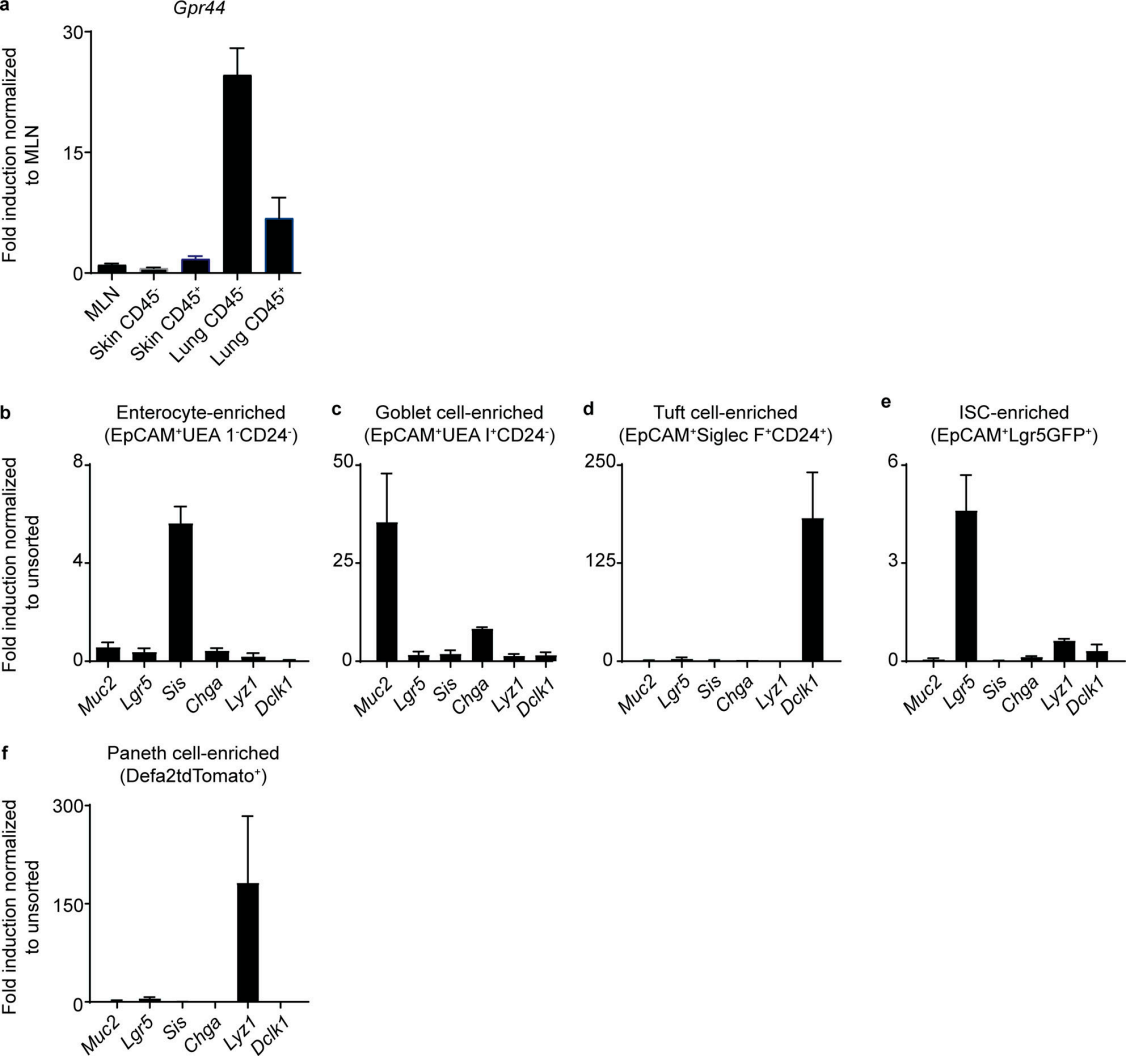
**Expression of *****Gpr44*****in skin and lungandlineage-associated genes in sorted IECs. (a)***Gpr44* expression in enriched CD45^+^ and CD45^−^ skin and lung fractions was determined using real-time PCR (MLN cells as positive control, calculated relative to *Actβ*). **(b–f)** Enrichment of expression of characteristic epithelial cell markers *Sis* for enterocytes, *Muc2* for goblet cells, *Dclk1* for tuft cells, *Lgr5* for ISCs, *Chga* for enteroendocrine cells, and *Lyz1* for Paneth cells by sorted enterocyte-enriched fractions gated as live, EpCAM^+^UEA 1*^−^*CD24*^−^* (b), sorted goblet cell–enriched fractions gated as live, CD45*^−^*EpCAM^+^UEA 1^+^CD24*^−^* (c), sorted tuft cell–enriched fractions gated as live, EpCAM^+^Siglec F^+^CD24^+^ (d), sorted ISC-enriched fractions gated as live, EpCAM^+^Lgr5GFP^+^ (e), and sorted Paneth cell–enriched fractions gated as Defa6tdTomato^+^ (f). Data are mean ± SEM. **(a)** *n* = 6; two independent experiments. **(b–f)** Five independent experiments from three or four pooled mice/experiment.

Expression of CRTH2 in murine IECs has not previously been characterized, perhaps due to the lack of a sensitive and specific anti-murine CRTH2 antibody. To elucidate if IECs express *Gpr44*, we first performed real-time quantitative PCR for *Gpr44* expression on a positive control tissue, the MLN, which is highly enriched for immune cells that can express CRTH2 (Oyesola et al., 2020b), and IECs isolated from the small intestine of WT and *Gpr44^−/−^* mice. We observed detectable levels of *Gpr44* in WT but not *Gpr44^−/−^* MLNs as well as IECs (Fig. 3 a). We also observed positive *Gpr44* expression in fractions enriched for skin and lung epithelial cells (Fig. S3 a). To confirm that the *Gpr44* signal in IECs was not a result of contaminating immune cells, we used a combination of flow cytometry and single-cell RNA transcript staining to identify that CD45^−^, EpCAM-expressing IECs expressed *Gpr44* (Fig. 3 b). These data show for the first time that murine IECs express *Gpr44* and have the capacity to respond to PGD_2_.

We next sought to investigate the factors that regulate *Gpr44* expression in murine IECs, measuring *Gpr44* expression in IECs isolated from the crypt or villi fractions of *N. brasiliensis*–infected compared with naive WT mice. In the steady state, *Gpr44* expression was enriched in the crypt compared with the villus fraction, and there was a specific infection-induced increase in *Gpr44* expression in the villus fraction (Fig. 3 c). Using an in vitro small intestinal organoid culture system that results in the generation of all known IEC lineages (Sato et al., 2009), stimulation of organoids with recombinant (rm)IL-4 and rmIL-13 increased *Gpr44* expression compared with media alone (Fig. 3 d), suggesting that Type 2 cytokine exposure drives *Gpr44* expression in murine small intestinal IECs.

Conversely, IFN-γ stimulation with a concentration below the threshold for IEC cytotoxicity (Farin et al., 2014) significantly decreased *Gpr44* expression in the presence or absence of concurrent rmIL-4 and rmIL-13 stimulation (Fig. 3 d). We also observed *GPR44* expression in human ascending colon mucosal biopsies from healthy individuals or those diagnosed with Crohn’s disease or ulcerative colitis, inflammatory bowel diseases associated with IFN-γ elevation (Neurath, 2014; Fig. 3 e). Consistent with a role for IFN-γ in reducing *GPR44* expression, samples from individuals with Crohn’s disease or ulcerative colitis had significantly decreased expression of *GPR44* in comparison to those from healthy controls, both in affected (inflamed) and adjacent unaffected tissues (Fig. 3 e). Together, these studies suggest that Type 1 and Type 2 cytokines likely play an antagonistic role in regulating *GPR44* expression by IECs in mice and humans.

### Stem, goblet, and tuft cell–enriched IEC fractions express *Gpr44*

**Figure 4.**
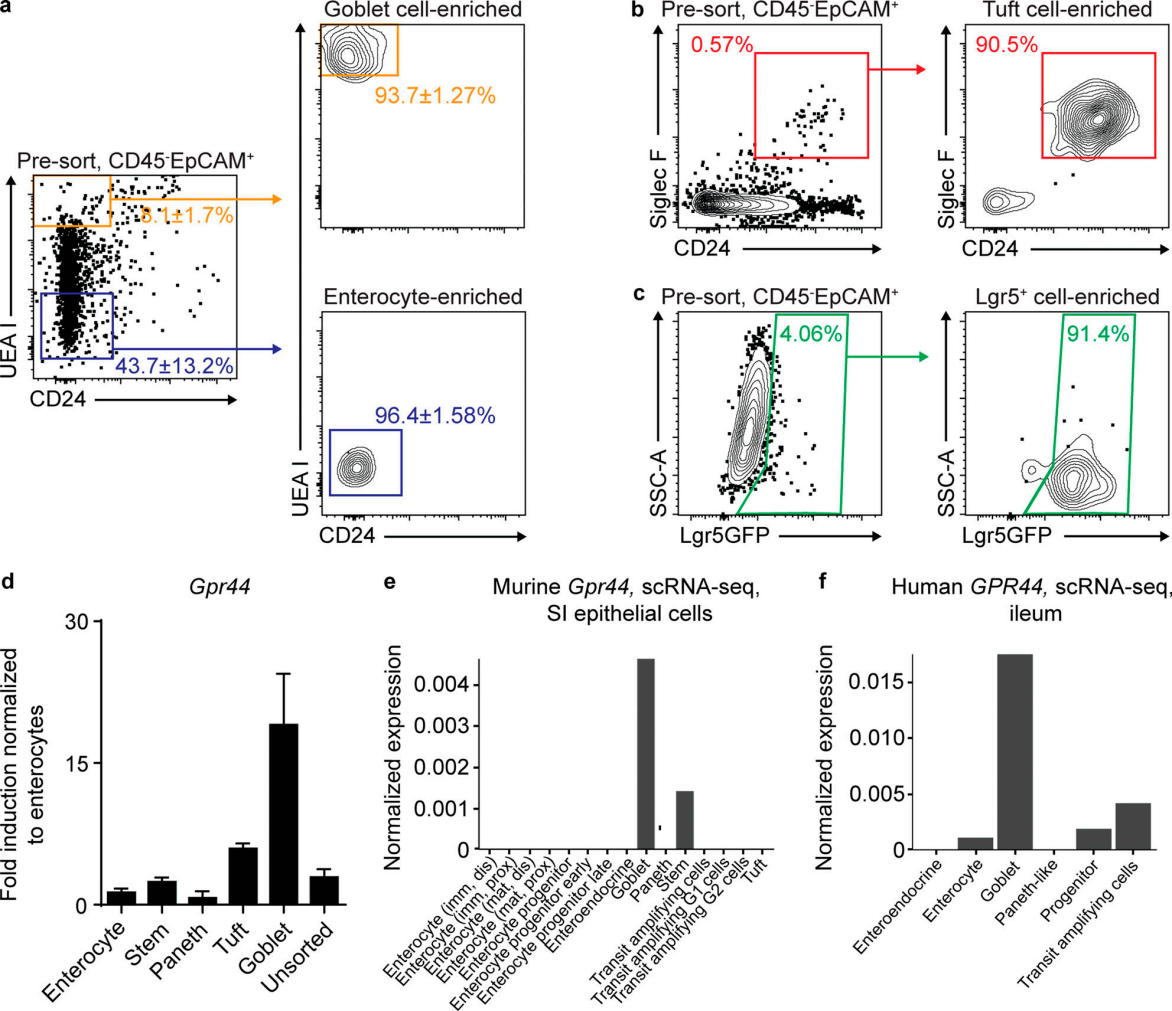
**ISC-, goblet cell–, and tuft cell–enriched IEC fractions express *Gpr44*.(a–c)** Naive (N) WT C57BL/6 small intestinal IECs were subjected to flow cytometry to identify fractions enriched for goblet cells (live, CD45*^−^*EpCAM^+^UEA 1^+^CD24*^−^*) and enterocytes (live, CD45*^−^*EpCAM^+^UEA 1*^−^*CD24*^−^*; a), tuft cell–enriched fractions (live, CD45*^−^*EpCAM^+^CD24^+^Siglec F^+^; b), and ISC-enriched fractions (live, CD45*^−^*EpCAM^+^Lgr5GFP^+^; c). **(d)** *Gpr44* expression in sorted IEC types was determined using real-time PCR, normalized to enterocytes. **(e and f)** Small intestinal (SI) IECs from N WT C57BL/6 mice (e) and ileum IECs from humans (f) were subjected to scRNA-seq to analyze *Gpr44*/*GPR44* expression in clusters (as in Haber et al. [2017] for mouse and Wang et al. [2020] for human). Data are mean ± SEM. **(a–c)** Representative plots, pooled from four different mice. **(d)** Three or four independent experiments from three or four pooled mice/experiment. **(e)** Described in Haber et al. (2017). **(f)** Described in Wang et al. (2020). dis, distal; imm, immature; mat, mature; prox, proximal; SSC-A, side scatter A.

Next, we sought to determine which small intestinal IEC lineages express *Gpr44*, hypothesizing that goblet and tuft cells and/or the ISCs that are the source of these lineages would express *Gpr44.* To test this hypothesis, we sort-purified various IEC lineages and used real-time PCR to identify *Gpr44*-expressing cell types. In enterocyte-enriched fractions (Fig. 4 a) that expressed *Sis* (Fig. S3 b), we observed detectable but quite low *Gpr44* expression (Fig. 4 d). Normalizing to this low expression level, we found that goblet cell–enriched fractions (Knoop et al., 2015; Nefzger et al., 2016; Smith et al., 2017; Wong et al., 2012; Fig. 4 a) that expressed *Muc2* (Fig. S3 c) were highly enriched for *Gpr44* expression (Fig. 4 d). Tuft cell–enriched fractions (Gerbe et al., 2016; Nadjsombati et al., 2018; Fig. 4 b) that expressed *Dclk1* (Fig. S3 d) also were enriched for *Gpr44* expression (Fig. 4 d). Lgr5GFP^+^ stem cells (Fig. 4 c and Fig. S3 e) also demonstrated *Gpr44* expression above that observed in enterocytes (Fig. 4 d). Paneth cell–enriched fractions (Fig. S3 f) did not show enrichment for *Gpr44* (Fig. 4 d). Consistent with these data, in analyzing published scRNA-seq datasets, we detected low levels of *Gpr44* expression in small numbers of goblet and stem cells from the murine small intestine (Haber et al., 2017; Fig. 4 e) and the human small intestine (Wang et al., 2020; Fig. 4 f). Tuft cell *Gpr44* expression was not detected, though this may be because of technical limitations of scRNA-seq. Thus, goblet and tuft cells and ISCs express *Gpr44*, supporting a role for the PGD_2_–CRTH2 pathway in regulating the accumulation and function of mature goblet and tuft cells and/or the ISCs from which they arise.

### Tuft cells are a source of PGD_2_ in the murine small intestine

**Figure 5.**
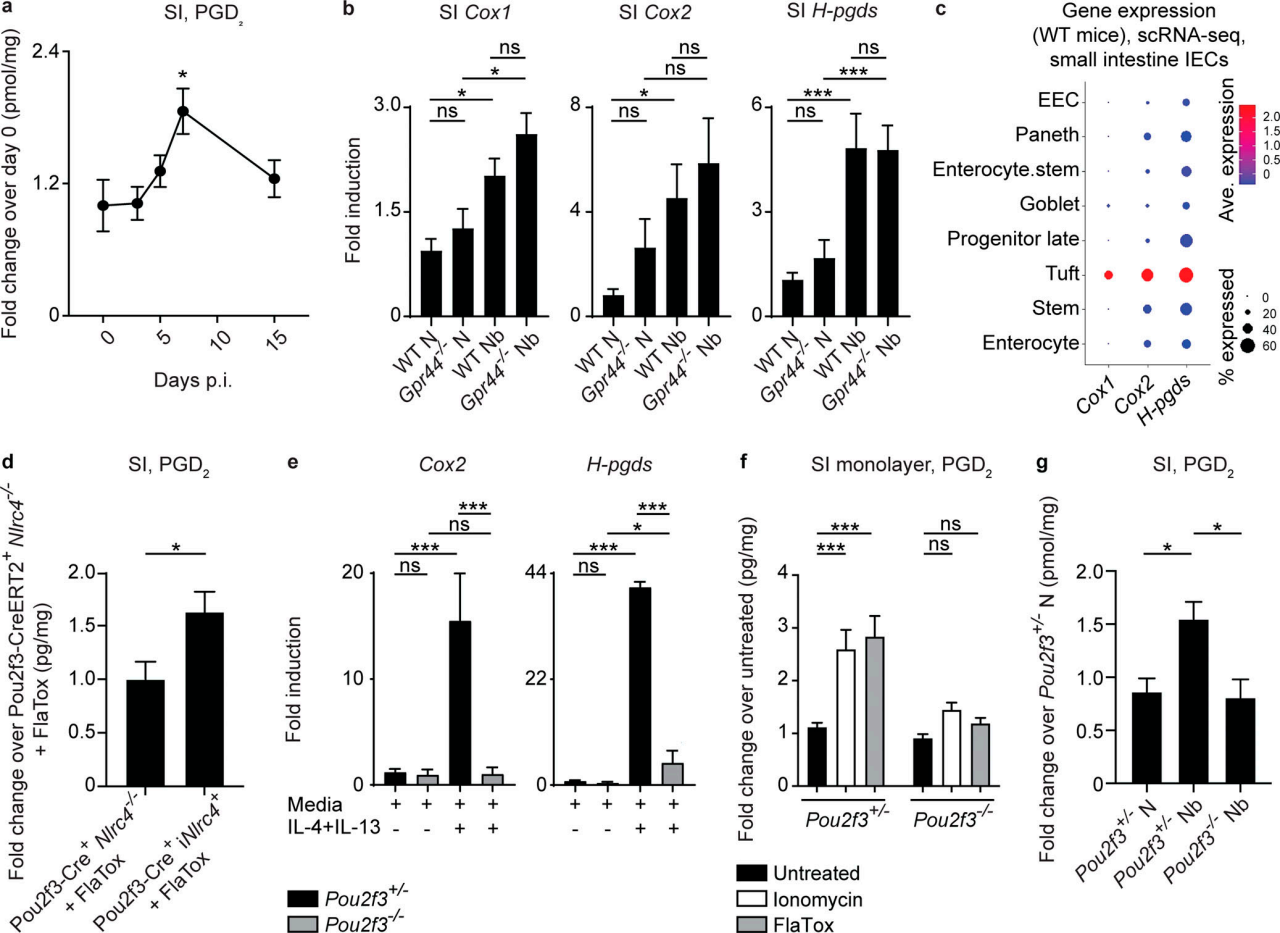
**Tuft cells are a source of PGD_2_ in the murine small intestine.(a)** On days 0, 3, 5, 7, and 15 p.i. in C57BL/6 WT mice infected with 500 *N. brasiliensis* (Nb) L3 larvae subcutaneously, PGD_2_ in small intestinal (SI) tissue homogenates/milligram of tissue was quantified by mass spectrometry (normalized to day 0). **(b)** On day 7 p.i. in littermate or cohoused C57BL/6 WT and *Gpr44^−/−^* mice infected with 500 Nb L3 larvae subcutaneously, levels of *Cox1*, *Cox2*, and *H-pgds* in small intestinal homogenates was measured by real-time PCR (relative to *Actβ* and normalized to WT naive [N]). **(c)** Small intestinal IECs from WT N mice were subjected to scRNA-seq to assess for *Cox1*, *Cox2*, and *H-pgds* expression in identified cell clusters. **(d)** PGD_2_ levels measured by mass spectrometry in jejunal homogenates/milligram of tissue of succinate- and tamoxifen-treated littermate C57BL/6 Pou2f3-CreERT2^+^ *Nlrc4^−/−^* or Pou2f3-CreERT2^+^ i*Nlrc4^+^* mice, all injected with 0.8 µg/g PA and 0.2 µg/g LFn-VP FlaA (FlaTox) for 20 min (normalized to succinate- and tamoxifen-treated Pou2f3-CreERT2^+^ *Nlrc4^−/−^* mice). **(e)** Small intestinal organoids from littermate C57BL/6 *Pou2f3^+/−^* or *Pou2f3^−/−^* mice were cultured for 2 d in media or Type 2 cytokines (250 µg/ml rmIL-4 and 250 µg/ml rmIL-13), and real-time PCR was used to measure expression of *Cox2* and *H-pgds*, calculated relative to *Gapdh*. **(f)** PGD_2_ levels measured by mass spectrometry in supernatants of intestinal monolayer cultures derived from mice of the indicated genotype following 30-min stimulation with 1 µg/ml ionomycin or 10 µg/ml PA + 5 µg/ml LFn-VP FlaA (FlaTox). **(g)** On day 7 p.i. in littermate C57BL/6 *Pou2^+/−^* and *Pou2f3^−/−^* mice infected with 500 Nb L3 larvae subcutaneously, PGD_2_ in small intestinal tissue homogenates/milligram of tissue was quantified by mass spectrometry (normalized to WT N mice). Data are mean ± SEM. **(a)** Analyzed using Dunnett’s test to identify differences between control time point (day 0) and every other time point. **(b, e, and g)** Analyzed using a linear mixed-effects model with pairwise comparison. **(d)** Analyzed using an unpaired Student’s *t* test. **(f)** Analyzed using a two-way ANOVA. **(a)** *n* = 4 mice/day of infection. **(b)** *Cox1* and *Cox2*, N, *n* = 5; Nb, *n* = 8 or 9. *H-pgds* N, *n* = 6 or 7; Nb, *n* = 11–13; three or four independent experiments. **(c)** Two independent experiments (one mouse in each group/experiment). **(d)** *n* = 4 or 5; one representative experiment of two independent experiments shown. **(e)** Three independent experiments. **(f)** *n* = 5 or 6/genotype and condition; two independent experiments. **(g)** *n* = 5–9; two independent experiments. Each *n* refers to number of mice/group in total across all experiments unless otherwise noted. *, P ≤ 0.05; ***, P ≤ 0.001. Ave., average; EEC, enteroendocrine cell.

**Figure S4.**
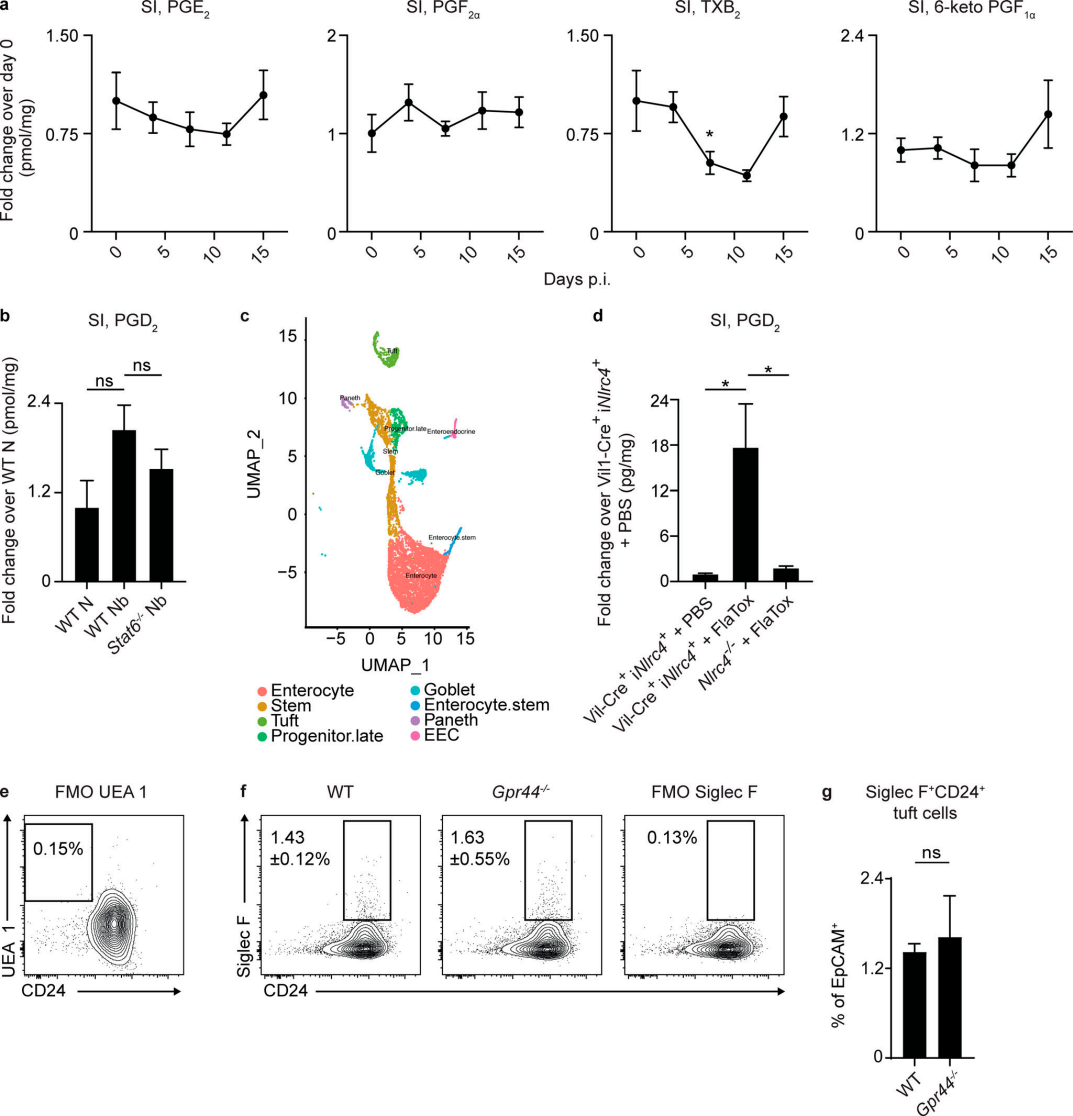
**Prostanoid responses in the small intestine and tuft cell responses in organoids.** Mice were infected with 500 *N. brasiliensis* (Nb) L3 larvae subcutaneously. **(a)** On days 3, 5, 7, and 15 p.i., the amounts of prostaglandins in small intestinal (SI) tissue homogenates/milligram of tissue were quantified using mass spectrometry in C57BL/6 WT mice (normalized to day 0). **(b)** On day 7 p.i., PGD_2_ in small intestinal tissue homogenates was quantified using mass spectrometry in WT and *Stat6^−/−^* mice (normalized to WT naive [N] mice). **(c)** Small intestinal IECs from WT N mice were subjected to scRNA-seq to generate UMAP plots of cell clusters following cluster assignment (as in Haber et al. [2017]). **(d)** PGD_2_ levels measured by mass spectrometry in ileal homogenates/milligram of tissue of *Nlrc4^−/−^* or Vil-Cre^+^ iNlrc4^+^ mice, injected with PBS or 0.8 µg/g PA and 0.2 µg/g LFn-VP FlaA (FlaTox) for 20 min (normalized to PBS-treated Vil-Cre^+^ iNlrc4^+^ mice). **(e)** Representative plot of fluorescence minus one (FMO) staining control for UEA 1 in organoid cultures. **(f and g)** Representative plots (f) and frequencies of tuft cells (live, CD45*^−^*EpCAM^+^Siglec F^+^CD24^+^; g) assessed using flow cytometry in organoids at day 10 after seeding; Siglec F FMO shown. Data are mean ± SEM except where noted. **(a)** Analyzed using Dunnett’s test to identify differences between control time point (day 0) and every other time point. **(b and d)** Analyzed using a one-way ANOVA. **(g)** Analyzed using a linear mixed-effects model with pairwise comparison. **(a)** *n* = 4 mice/day of infection. **(b)** *n* = 2–4 mice/group; one experiment. **(c)** Two independent experiments (one mouse in each group/experiment). **(d)** *n* = 6 or 7; two independent experiments. **(e–g)** Two independent experiments. *, P ≤ 0.05. EEC, enteroendocrine cell.

The identification of CRTH2 expression in IECs drove us to hypothesize that local intestinal cell types might produce PGD_2_, as prostaglandins are highly labile factors that likely do not diffuse far from the site where they are produced (Harris et al., 2002; Serhan et al., 2015). Recent data have suggested that tuft cells can produce PGD_2_ (Bezençon et al., 2008; DelGiorno et al., 2020; Gerbe et al., 2016; Haber et al., 2017), provoking the hypothesis that tuft cells are a source of PGD_2_ that could act on IECs during helminth infection. On day 7 p.i., coincident with the peak of *N. brasiliensis*–induced tuft cell hyperplasia (Gerbe et al., 2016; von Moltke et al., 2016), there was a significant infection-induced increase in PGD_2_ (Fig. 5 a), but not other prostaglandins or thromboxane (Fig. S4 a), in small intestinal tissue detected by mass spectrometry. There was a trend toward decreased infection-induced PGD_2_ in *Stat6^−/−^* mice (Fig. S4 b). At day 7 p.i. in small intestinal homogenates, there was an increase in the expression of genes encoding for enzymes in the PGD_2_ synthesis cascade including *Cox1*, *Cox2*, and *H-pgds* (hematopoietic PGD_2_ synthase; Fig. 5 b). Further, in our scRNA-seq of naive WT small intestinal IECs (Fig. S4 c), the majority of tuft cells highly expressed *Cox1*, *Cox2*, and *H-pgds*, with no other epithelial lineage significantly enriched for expression of all the enzymes required for PGD_2_ synthesis (Haber et al., 2017; Fig. 5 c).

To test whether tuft cells can produce PGD_2_ in the small intestine in vivo following stimulation, we used a genetic approach that allowed for the specific activation of tuft cells. Mice that express NLRC4 only in epithelial or tuft cells were treated with FlaTox, which delivers the NAIP5 ligand flagellin to the cytosol to activate the NAIP5-NLRC4 inflammasome, a powerful stimulus of prostaglandin production (Ballard et al., 1996; Rauch et al., 2017; von Moltke et al., 2012). FlaTox-treated Vil-Cre^+^ i*Nlrc4*^+^ mice that only express *Nlrc4* in IECs had significantly higher small intestinal PGD_2_ levels, as measured by mass spectrometry, compared with PBS-treated Vil-Cre^+^ i*Nlrc4*^+^ or FlaTox-treated *Nlrc4^−/−^* controls (Fig. S4 d). Following succinate treatment to specifically activate and expand tuft cells and subsequent FlaTox treatment, Pou2f3-CreERT2^+^ i*Nlrc4*^+^ mice that only express *Nlrc4* in tuft cells showed a significant increase in small intestinal PGD_2_ levels measured by mass spectrometry compared with FlaTox-treated *Nlrc4^−/−^* controls (Fig. 5 d). These data suggest that among IECs, tuft cells are specifically capable of producing PGD_2_ in response to a known stimulator of prostaglandin production.

We next tested whether tuft cells were required for epithelial production of PGD_2_ in response to Type 2 inflammatory signals. When tuft cell–sufficient and –deficient (Gerbe et al., 2016) small intestinal organoids were treated with rmIL-4 and rmIL-13, control but not tuft cell–deficient organoids up-regulated *Cox2* and *H-pgds* compared with media alone (Fig. 5 e). Further, there was a significant increase in PGD_2_ levels in supernatants from ex vivo IEC monolayers from WT but not tuft cell–deficient mice treated with the PGD_2_-inducing stimulant ionomycin or FlaTox (Ballard et al., 1996; Rauch et al., 2017; von Moltke et al., 2012; Fig. 5 f). Finally, in vivo, *Pou2f3^−/−^* mice had significantly lower levels of PGD_2_ than tuft cell–sufficient mice in the small intestinal homogenates as measured by mass spectrometry on day 7 p.i. with *N. brasiliensis* (Fig. 5 g). Altogether, these in vitro, ex vivo, and in vivo approaches highlight that tuft cells are likely a critical source of PGD_2_ in the small intestine that locally regulates IEC responses during helminth infection.

### CRTH2-deficient murine small intestinal organoids display enhanced budding and goblet cell accumulation in the absence of Type 2 cytokines

**Figure 6.**
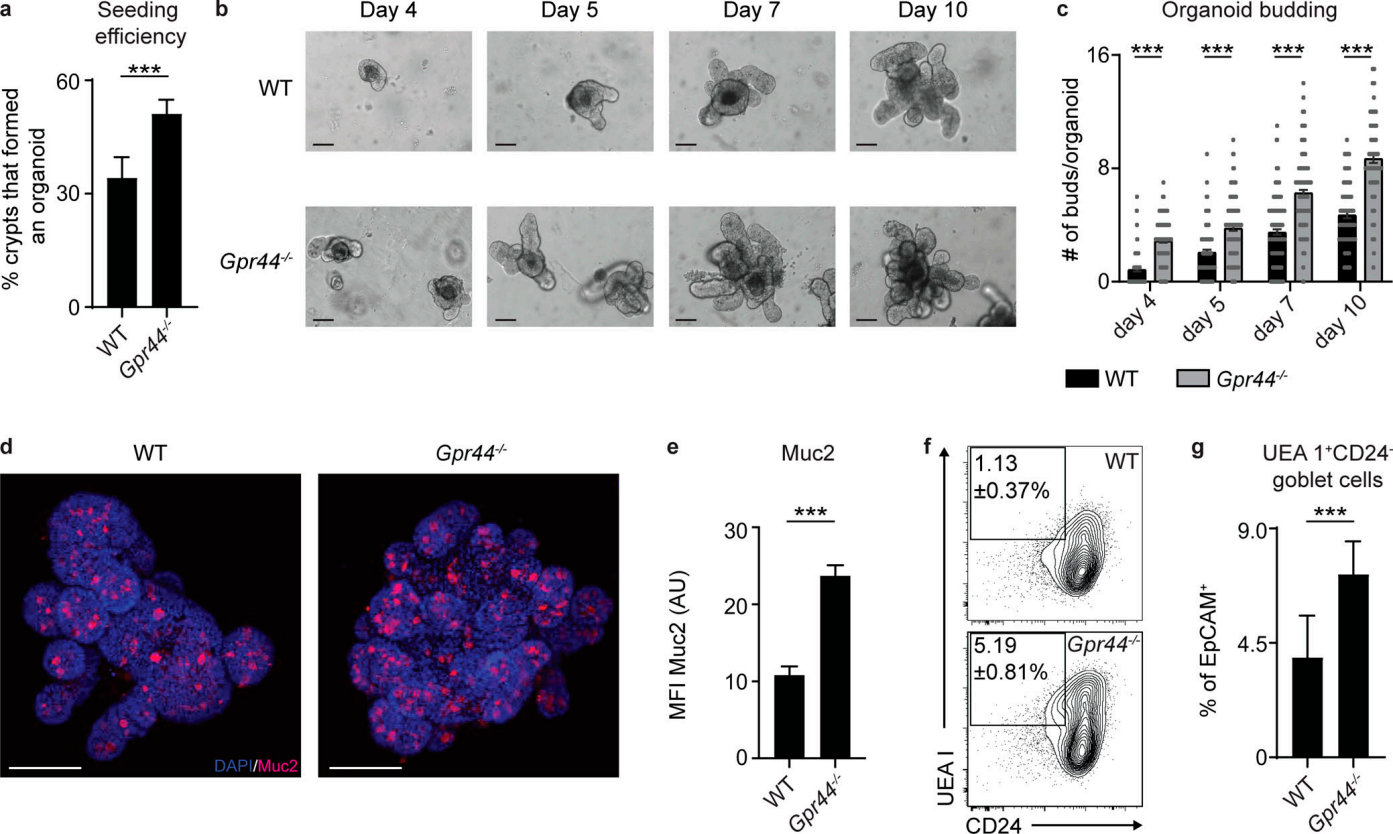
**CRTH2 limits murine small intestinal organoid budding and goblet cell accumulation.(a–c)** Small intestinal organoids from littermate C57BL/6 WT and *Gpr44^−/−^* mice were cultured and assessed directly after seeding. **(a)** Organoids were assessed for seeding efficiency at day 7 after seeding (calculated as number of organoids/well/input number of crypts, where the input number was equivalent for WT and *Gpr44^−/−^* genotypes). **(b and c)** Representative light microscopy images on days 4, 5, 7, and 10 after seeding (b) were quantified for the number of buds/organoid in ∼50 organoids/genotype (c). Scale bar = 200 µm. **(d and e)** Representative images showing immunofluorescence staining for Muc2 (red) and DAPI (blue) in whole-mount organoids on day 10 after seeding (d) were quantified for Muc2 MFI (AU; e). Scale bar = 100 µm. **(f and g)** Representative plots (f) and frequencies of goblet cells (live, CD45*^−^*EpCAM^+^UEA 1^+^CD24*^−^*; g) assessed using flow cytometry in organoids at day 10 after seeding. Data are mean ± SEM. **(a and g)** Analyzed using an unpaired Student’s *t* test (only one value/experiment). **(c and e)** Analyzed using a linear mixed-effects model with pairwise comparison. **(a–g)** Three experiments with independent organoid lines for WT and *Gpr44^−/−^* organoids. **(c and e)** 15–20 organoids/genotype/experiment analyzed. ***, P ≤ 0.001.

To focus on the mechanism by which the PGD_2_–CRTH2 pathway regulates IEC responses, we further employed the in vitro small intestinal organoid model. The differentiation of ISCs to the secretory lineages is a critical feature of the Type 2 cytokine–induced response that promotes worm clearance (Allen and Maizels, 2011; Coakley and Harris, 2020; Oyesola et al., 2020b; Pulendran and Artis, 2012). Since ISCs expressed *Gpr44* (Fig. 4) and *Gpr44^−/−^* mice had elevated goblet and tuft cell numbers (Fig. 2), we hypothesized that the PGD_2_–CRTH2 pathway might limit the growth potential of ISCs as well as differentiation into these cell lineages. Consistent with this hypothesis, we found that crypts freshly isolated from *Gpr44^−/−^* mice displayed an increased capacity to form an organoid in standard culture conditions compared with WT crypts (Fig. 6 a), and *Gpr44^−/−^* organoids formed more buds on days 4–10 after seeding, suggesting enhanced differentiation and growth (Fig. 6, b and c). Mirroring the enhanced goblet cell accumulation we observed in vivo in *Gpr44^−/−^* mice (Fig. 2), *Gpr44^−/−^*compared with WT organoids had increased Muc2 staining by immunofluorescence (Fig. 6, d and e) and higher frequencies of IECs that fell in the goblet (Fig. 6, f and g; and Fig. S4 e) but not tuft cell (Fig. S4, f and g) lineage by flow cytometry. Thus, organoids formed ex vivo from naive CRTH2-deficient mice have enhanced budding and goblet cell accumulation, all in the absence of Type 2 cytokine stimulation, suggesting that CRTH2 directly suppresses terminal differentiation of IECs toward the goblet cell fate.

### The PGD_2_–CRTH2 pathway counteracts Type 2 cytokine–elicited changes in IEC function

Helminth infection has a wide array of effects on IECs, reshaping the Lgr5^+^ compartment, increasing IEC proliferation, inducing villus atrophy and crypt hyperplasia, increasing the pace at which cells move through the epithelial escalator, and skewing to secretory lineage differentiation, all components of the weep and sweep response that expels helminths (Artis and Grencis, 2008; Cliffe et al., 2005; Gerbe et al., 2016; Hasnain et al., 2012; Howitt et al., 2016; McDermott et al., 2005; McKenzie et al., 1998; Nusse et al., 2018; von Moltke et al., 2016). Type 2 cytokines alone have overlapping and also discreet effects, depleting Lgr5^+^ cells and limiting the normal turnover of the epithelium to support emergence of differentiated lineages (Biton et al., 2018; Nusse et al., 2018). Based on our in vivo and in vitro data (Fig. 2 and Fig. 6), we hypothesized that the PGD_2_–CRTH2 pathway may regulate IEC proliferation, the rate at which cells move through the epithelial escalator, and goblet cell accumulation in response to Type 2 cytokines.

**Figure 7.**
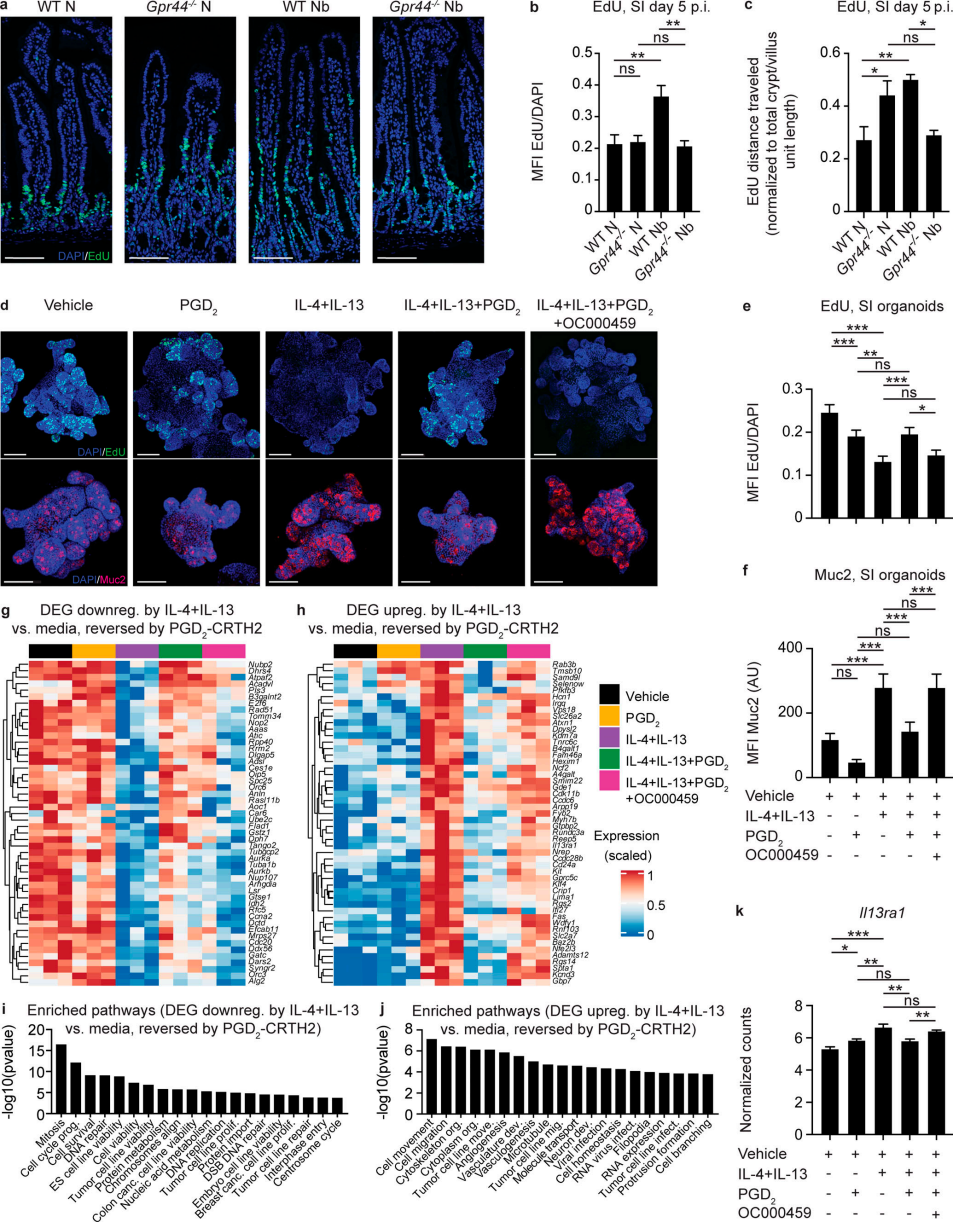
**The PGD_2_-CRTH2 pathway limits the Type 2 cytokine–induced IEC functional program.(a–c)** Littermate or cohoused C57BL/6 WT and *Gpr44^−/−^* mice were infected with 500 *N. brasiliensis* (Nb) L3 larvae subcutaneously and treated i.p. with EdU on day 4 p.i. In naive (N) mice and mice on day 5 p.i., representative images showing immunofluorescent staining for EdU in histological sections of the small intestine (SI; a) were quantified for the ratio of the EdU (green) MFI to DAPI (blue) MFI (b) and the distance EdU^+^ cells had traveled in the crypt/villus unit normalized to the total crypt/villus length (c). Scale bar = 100 µm. Small intestinal organoids from C57BL/6 WT mice were cultured for 2–3 d in media or Type 2 cytokines (250 µg/ml rmIL-4 and 250 µg/ml rm IL-13) with or without 20 µM PGD_2_ and with or without 1 µM OC000459 (CRTH2 inhibitor). **(d–f)** Representative images showing immunofluorescence staining for EdU (green, upper row) or Muc2 (red, lower row) and DAPI (blue) in whole-mount organoids (d) were quantified for the EdU ratio to DAPI MFI (e) or Muc2 MFI (f). Scale bar = 100 µm. **(g and h)** Organoids were dissociated, sort-purified for live cells, and subjected to RNA-seq to identify DEGs that were down-regulated (downreg.; g) or up-regulated (upreg.; h) by culture with rmIL-4+rmIL-13 compared with vehicle alone and reversed when PGD_2_ was added, in a CRTH2-dependent manner (top 50 displayed). **(i and j)** IPA was used to identify functional pathways that were enriched in these DEG sets, showing the top 20 ordered by P value of those with a z-score >2.0 (up-regulated) or less than −2.0 (down-regulated). **(k)** *Il13ra1* expression (normalized counts) in organoids from RNA-seq data. Data are mean ± SEM. **(b, c, e, and f)** Analyzed using a linear mixed-effects model with pairwise comparison. **(k)** Analyzed using a one-way ANOVA. **(a–c)** N, *n* = 4 or 5; Nb, *n* = 4 or 5 (5–10 crypt/villus units were examined/mouse and those values were averaged); two independent experiments. **(d–f)** *n* = 5–10 organoids/condition/experiment; four independent experiments. **(g–k)** *n* = 3 replicates/condition; one experiment. Each *n* refers to number of mice/group in total across all experiments unless otherwise noted. *, P ≤ 0.05; **, P ≤ 0.01, ***, P ≤ 0.001. canc., cancer; dev., development; DSB, double-stranded break; ES, embryonic stem; infect., infection; mig., migration; move., movement; org., organization; prolif., proliferation; prog., progression.

**Figure S5.**
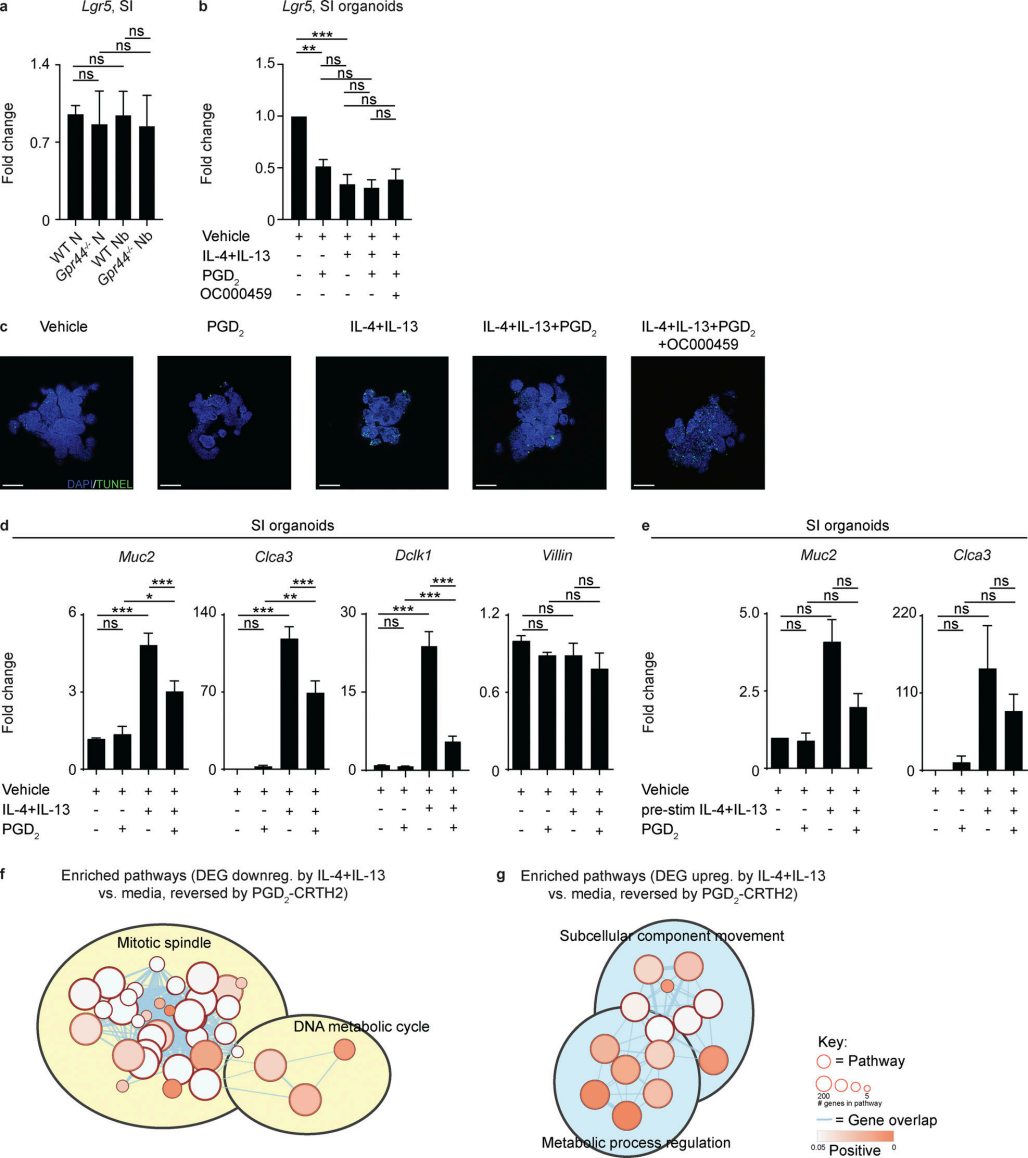
***Lgr5* expression in WT and *Gpr44^−/−^* mice following *N. brasiliensis* infection and organoid TUNEL staining and gene expression in response to Type 2 cytokines with or without engagement of the PGD_2_-CRTH2 pathway. (a)** C57BL/6 WT and *Gpr44^−/−^* mice were infected with 500 *N. brasiliensis* (Nb) L3 larvae subcutaneously, and the expression of *Lgr5* in small intestinal (SI) homogenates was measured by real-time PCR (relative to *Actβ* and normalized to WT naive [N]). Small intestinal organoids from C57BL/6 WT mice were cultured for 2 d in media or Type 2 cytokines (250 µg/ml rmIL-4 and 250 µg/ml rmIL-13) with or without 20 µM of PGD_2_ and with or without 1 µM OC000459. **(b)** Real-time PCR was used to measure expression of *Lgr5* calculated relative to *Gapdh*. **(c)** Representative images showing immunofluorescence staining for TUNEL in whole-mount organoids. Scale bar = 100 µm. **(d)** Real-time PCR was used to measure expression of *Muc2*, *Clca3*, *Dclk1*, and *Villin* calculated relative to *Gapdh*. **(e)** Organoids were prestimulated (pre-stim) with Type 2 cytokines with or without 20 µM PGD_2_. Real-time PCR was used to measure expression of *Muc2* and *Clca3*, calculated relative to *Gapdh*. **(f and g)** Enrichment map of functional pathways associated with PGD_2_-responsive genes that were down-regulated (downreg.; f) or up-regulated (upreg.; g) by IL-4+IL-13 treatment of organoids. Related pathways were clustered and annotated in Cytoscape. Data are mean ± SEM. **(a, b, d, and e)** Analyzed using a linear mixed-effects model with pairwise comparison. **(a)** N, *n* = 10–12; Nb, *n* = 14–19; six independent experiments. **(b)** Three or four experiments. **(c)** Two experiments. **(d and e)** Three to six experiments. **(f and g)** *n* = 3 replicates/condition; one experiment. Each *n* refers to number of mice/group in total across all experiments unless otherwise noted. *, P ≤ 0.05; **, P ≤ 0.01; ***, P ≤ 0.001.

To test this hypothesis, we first examined IEC proliferation and movement along the epithelial escalator in vivo using 5-ethynyl-2′-deoxyuridine (EdU) labeling in WT and *Gpr44^−/−^* mice infected with *N. brasiliensis*. At day 5 p.i. (when parasite burdens are comparable; Fig. 1 a), there was an infection-induced increase in total EdU signal normalized to total DNA staining (DAPI), suggesting that infected WT mice had more proliferating IECs than *Gpr44^−/−^* mice (Fig. 7, a and b). In addition, there was an infection-induced increase in the distance EdU^+^ cells had traveled along the crypt–villus axis normalized to crypt/villus length (Artis and Grencis, 2008; McDermott et al., 2005) in WT but not *Gpr44^−/−^* mice, suggesting that IECs in WT infected mice had an increased rate of movement through the epithelial escalator than in *Gpr44^−/−^* mice (Fig. 7, a and c). Expression of *Lgr5* in the small intestinal tissue was similar in naive and infected *Gpr44^−/−^* and WT mice at day 7 p.i. (Fig. S5 a). In the naive state, there was no difference in EdU signal normalized to DAPI between WT and *Gpr44^−/−^* mice, but there was an increase in the distance EdU^+^ cells had traveled in *Gpr44^−/−^* mice (Fig. 7, a–c). Thus, during infection, WT mice that have Type 2 cytokines and an operational PGD_2_–CRTH2 pathway had elevated IEC proliferation and rate of cell movement through the escalator following infection compared with *Gpr44^−/−^* mice that have Type 2 cytokines but lack the PGD_2_–CRTH2 axis. These data suggest that in a Type 2 cytokine–rich environment, CRTH2 promotes IEC proliferation and cell movement through the epithelial escalator.

To further explore this idea in the absence of potentially confounding effects from CRTH2-expressing immune cells and helminths, we treated murine small intestinal organoids with Type 2 cytokines and measured EdU labeling in the presence or absence of PGD_2_ and a specific CRTH2 inhibitor, OC000459. Organoids treated with Type 2 cytokines alone showed a substantial decrease in EdU signal normalized to DAPI (Fig. 7, d and e), associated with decreased *Lgr5* expression (Fig. S5 b). These findings are consistent with what was shown previously when IECs were exposed to Type 2 cytokines in the absence of other factors such as PGD_2_ and a pathogen (Biton et al., 2018). The decrease in EdU labeling rebounded with the addition of PGD_2_ in a CRTH2-dependent manner (Fig. 7, d and e). This finding mimics our observations in vivo comparing infected WT mice (Type 2 cytokines + PGD_2_ acting via CRTH2) versus *Gpr44^−/−^* mice (Type 2 cytokines + PGD_2_ without CRTH2; Fig. 7, a and b). The decrease in *Lgr5* expression was not PGD_2_–CRTH2 dependent (Fig. S5 b). Apoptosis, as measured using Tdt-mediated dUTP-biotin nick end labeling (TUNEL) staining, did not appreciably change across conditions (Fig. S5 c). Type 2 cytokines also elicited increased Muc2 staining (Fig. 7, d and f) and expression of goblet and tuft cell–associated genes, including following pretreatment with Type 2 cytokines (Fig. S5, d and e). Increased Muc2 staining was reversed with PGD_2_ treatment, dependent on CRTH2 (Fig. 7, d and f), again mirroring what we observed in vivo in WT and *Gpr44^−/−^* mice (Fig. 2, d and e). Together, these data illustrate that Type 2 cytokine exposure causes decreased IEC proliferation and increased goblet cell emergence and that the PGD_2_–CRTH2 pathway counteracts this program.

**Figure 8.**
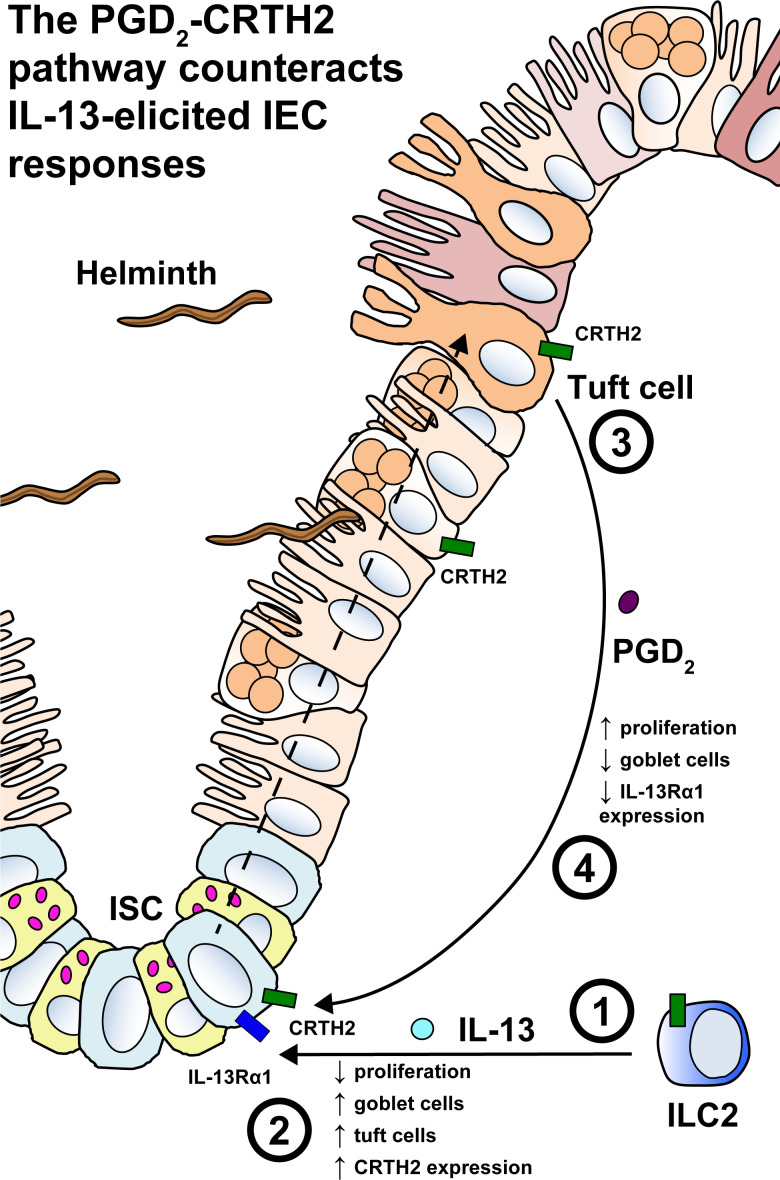
**The PGD_2_–CRTH2 pathway counteracts the effect of Type 2 cytokines on the small intestinal epithelium during helminth infection.** (1) Infection with intestinal helminth parasites elicits IL-13 production from ILC2s and other immune cell types that produce IL-13. (2) IL-13 acts on IL-13Rα1–expressing ISCs and other IECs, causing decreased proliferation, terminal differentiation, loss of Lgr5-expressing stem cells, goblet cell hyperplasia, and tuft cell hyperplasia. CRTH2 expression is also up-regulated. (3 and 4) Increased tuft cells are activated to produce PGD_2_ (3), which then acts back on CRTH2-expressing ISCs, and possibly other epithelial cell types that express CRTH2 (not depicted), to counteract the effects of IL-13 (4), increasing proliferation, suppressing goblet cell accumulation, and decreasing IL-13Rα1 expression.

To delve further into the PGD_2_-CRTH2–dependent regulation of the IEC response to Type 2 cytokines, we performed RNA sequencing (RNA-seq) of organoid cells treated with Type 2 cytokines in the presence or absence of PGD_2_ and OC000459. We identified suites of genes that were either up- or down-regulated by IL-4+IL-13 compared with media alone and that were responsive to PGD_2_ via CRTH2, leading to a reversal in IL-4+IL-13–induced expression patterns (Fig. 7, g and h). Functional pathway analysis using Ingenuity Pathway Analysis (IPA) on these differentially expressed genes (DEGs) revealed that Type 2 cytokines decreased the expression of genes associated with cell division and DNA replication pathways and that addition of PGD_2_ reversed this effect in a CRTH2-dependent manner (Fig. 7 i). Type 2 cytokines increased the expression of genes associated with cell movement, branching, and cytoskeletal rearrangement, all pathways associated with terminal differentiation, again with PGD_2_ reversing this effect in a CRTH2-dependent manner (Fig. 7 j; and Fig. S5, f and g). Critically, one of the genes up-regulated by IL-4+IL-13 was *Il13ra1*, which was then down-regulated by PGD_2_, dependent on CRTH2 (Fig. 7 k), suggesting that the PGD_2_–CRTH2 pathway can directly oppose the IL-13–mediated transcriptional program in organoid IECs via receptor down-regulation. Together, our data show that in the presence of Type 2 cytokines, the PGD_2_–CRTH2 pathway promotes epithelial proliferation and puts a brake on terminal differentiation to the goblet cell lineage, counteracting the Type 2 cytokine–elicited IEC response (Fig. 8).

## Discussion

Helminth infection promotes increased epithelial cell proliferation and movement through the epithelial escalator, increased intestinal permeability, and goblet and tuft cell hyperplasia that are essential for effective worm expulsion. These changes must be tightly regulated to prevent immunopathology and a return to homeostasis (Allen and Maizels, 2011; Coakley and Harris, 2020; Oyesola et al., 2020b; Pulendran and Artis, 2012). While hallmark studies have revealed a key role for cytokines in modulating helminth-induced epithelial changes (Gieseck et al., 2018; Lindholm et al., 2020* Preprint*) and eicosanoids such as lipoxins and resolvins resolve inflammation (Serhan et al., 2015), the role of prostaglandins, specifically PGD_2_ (de los Reyes Jiménez et al., 2020), in the modulation of Type 2 cytokine–induced epithelial cell responses is less clear. In this study, we demonstrate a previously unappreciated role for PGD_2_ and its receptor CRTH2 in dampening Type 2 cytokine–induced IEC responses.

Eicosanoid levels increase during Type 2 inflammation in mice and humans (Oyesola et al., 2020b; Fig. 5 a), but the relevant cellular source of PGD_2_ in tissues was previously unclear. Various cell types, including immune and other hematopoietic cells (Lewis et al., 1982; MacDermot et al., 1984; Oelz et al., 1977; Tanaka et al., 2000), have the ability to produce eicosanoids. Mast cells were implicated as a rich source of prostaglandins (Heavey et al., 1988), but mast cell deletion studies in hookworm infection have yielded variable results (Hepworth et al., 2012; Mukai et al., 2016; Newlands et al., 1995; Ohnmacht and Voehringer, 2010). Recent work has identified tuft cells as a key source of leukotrienes (McGinty et al., 2020), and tuft cells have the capacity to produce PGD_2_ (Bezençon et al., 2008; DelGiorno et al., 2020; Gerbe et al., 2016; Haber et al., 2017; Fig. 5 c). We found that when tuft cells are the only epithelial cells that can respond to a stimulus that promotes prostaglandin synthesis, PGD_2_ levels increase (Fig. 5 d), and epithelial monolayers and mice that lack tuft cells are deficient in their ability to produce PGD_2_ following stimulation (Fig. 5, f and g). Thus, as PGD_2_ is a labile factor that likely acts locally (Harris et al., 2002; Serhan et al., 2015), tuft cells may serve as a key local source of PGD_2_ that shapes the IEC response during helminth infection (Fig. 8). Further studies that measure PGD_2_ in vivo in the absence of tuft cell capacity to make PGD_2_ will be required to determine if tuft cells are necessary to produce normal, infection-induced PGD_2_ levels.

Helminth infection and Type 2 cytokines (specifically IL-13) both regulate IEC functions, but the effects of helminth infection are not precisely the same as those of IL-13. For example, a fetal-like state emerges in ISCs that are near granulomas during *Heligmosomoides polygyrus* infection (Nusse et al., 2018), and helminth infection increases IEC proliferation and the rate at which cells move through the epithelial escalator (Cliffe et al., 2005). IL-13 alone in the absence of infection results in a loss of stemness and decreased proliferative capacity and the emergence of a highly differentiated trajectory of IECs, including goblet and tuft cells (Biton et al., 2018; Gerbe et al., 2016; Hasnain et al., 2012; Howitt et al., 2016; McKenzie et al., 1998; von Moltke et al., 2016). Helminths manipulate the host response in many ways. As such, helminth excretory-secretory products that modulate the epithelium directly or indirectly may engage with the PGD_2_–CRTH2 pathway and also may help to explain the differences between helminth- and IL-13–elicited IEC responses (Maizels et al., 2018). Our in vivo and in vitro EdU labeling experiments also in part address this discrepancy. Our results show that *Gpr44^−/−^* mice have decreased proliferation in vivo following helminth infection (Fig. 7, a and b) and that PGD_2_ via CRTH2 counteracts IL-13–mediated repression of IEC proliferation in vitro (Fig. 7, d and e). These data suggest that the PGD_2_–CRTH2 pathway potentially protects the proliferative capacity of the ISC pool and promotes increased epithelial movement through the escalator that helps to expel worms (Cliffe et al., 2005). Of note, while *Gpr44^−/−^* compared with WT mice had defective movement of EdU-labeled cells up the crypt–villus axis after *N. brasiliensis* infection (Fig. 7, a and c), they still demonstrated enhanced worm clearance. This may be due to increased goblet cell accumulation, but future studies are required to dissect this finding. Importantly, *Gpr44* expression and PGD_2_ production are up-regulated by Type 2 cytokines (Fig. 3 and Fig. 5), suggesting that IL-13 engages the PGD_2_–CRTH2 pathway to toggle its own effect on IECs, dictating the balance between parasite expulsion and maintenance of the IEC layer (Fig. 8).

In addition, our data also highlight a role for CRTH2 in regulating IEC responses in the absence of Type 2 cytokines. We observed effects of CRTH2 deficiency on organoid budding and goblet cell accumulation in normal media conditions (Fig. 6). In these cultures, there may be endogenous PGD_2_ made by tuft cells that affects terminal differentiation, although it is also possible that ISCs in CRTH2-deficient mice are programmed in vivo and this program persists in vitro. Crossing *Gpr44^−/−^* mice with *Pou2f3^−/−^* or *Il4ra^−/−^* mice and assessing organoid budding and goblet cell accumulation would help to dissect this effect further. We also found that naive *Gpr44^−/−^* mice had an increased rate of IEC movement through the epithelial escalator compared with naive WT controls (Fig. 7, a and c), though overall IEC proliferation was similar (Fig. 7, a and b). This result could point to effects of the PGD_2_–CRTH2 pathway on cell movement that are independent of proliferation. There are likely distinct mechanisms that govern the effect of the PGD_2_–CRTH2 pathway on IECs and immune cells in the steady state and during Type 2 inflammation.

Mechanistically, PGD_2_ and CRTH2 may limit IEC, specifically ISC, responses to Type 2 cytokines by suppressing the ability to respond to IL-13 by down-regulating *Il13ra1* (Fig. 7 k). However, in our RNA-seq experiment, decreased *Il13ra1* expression could be due to a decline in the frequency of the cell types that express *Il13ra1*. Our RNA-seq data show that the PGD_2_–CRTH2 pathway regulates genes that control an array of IEC functions, ranging from metabolism to cytoskeletal organization. Thus, there may be other mechanisms by which the PGD_2_–CRTH2 pathway counteracts the Type 2 cytokine–elicited program in IECs. In particular, CRTH2 expression by tuft and goblet cells (Fig. 4 d) may have direct consequences for the function or survival of these cells during Type 2 inflammation. Further work characterizing in vivo and in vitro systems that allow for goblet cell– or tuft cell–specific deletion of *Gpr44* will be needed to elucidate the significance of mature goblet and tuft cell CRTH2 expression.

Finally, there is more work to be done to understand the pro- versus anti-inflammatory properties of the PGD_2_–CRTH2 pathway. CRTH2 plays a clear pro-inflammatory role in promoting immune cell function (Oyesola et al., 2020b). While we did not find a marked deficit in immune cell population expansion in *Gpr44^−/−^* compared with WT mice (Fig. 1, b–d; and Fig. S1, a–g), further investigation of immune responses in the intestine in CRTH2-deficient mice is warranted. Regardless, as the PGD_2_–CRTH2 pathway has both pro- and anti-inflammatory effects, temporal or spatial regulation of PGD_2_ production or CRTH2 expression may be at play to successfully expel the parasite and balance Type 2 cytokine–induced IEC changes. Ultimately, CRTH2-floxed mice will be needed to fully understand the effects of CRTH2 on IECs and immune cells in the intestine, lung, and skin and during chronic diseases or infections, including during chronic or trickle infection with *H. polygyrus* (Colombo and Grencis, 2020). Additional studies will also be required to test how CRTH2 deficiency during helminth infection impacts the return to intestinal homeostasis and response to subsequent injury or infection. Such investigations will be needed to determine whether and how the PGD_2_–CRTH2 pathway confers advantage to the host during and after helminth infection.

Thus, we show that the PGD_2_–CRTH2 pathway limits Type 2 cytokine–mediated decreases in proliferation and increases in terminal differentiation in small intestinal IECs (Fig. 8). These data inform efforts to manipulate this pathway in the treatment of Type 2 inflammatory diseases in humans. This function also has clear implications for the outcome of helminth infection, with the PGD_2_–CRTH2 pathway potentially tuning the intestinal milieu to expel worms while maintaining key homeostatic functions. Together, our data highlight the importance of the regulation of the Type 2 cytokine–elicited IEC functional program, revealing that the PGD_2_–CRTH2 pathway is a critical player in shaping IEC responses during Type 2 inflammation.

## Materials and methods

### Mice

Male and female C57BL/6 WT 45.1 and CD45.2 mice were purchased from The Jackson Laboratory or bred in-house. *Stat6^+/+^* and *Stat6^−/−^* mice were bred in-house. *Gpr44^−/−^* mice were provided by Amgen Inc. *Pou2f3^−/−^* and littermate *Pou2f^+/−^* mice were provided by J. von Moltke. Vil-Cre^+^ i*Nlrc4*^+^, *Nlrc4^−/−^*, Pou2f3-CreERT2^+^ i*Nlrc4*^+^, and Pou2f3-CreERT2^+^ *Nlrc4^−/−^* mice were provided by I. Rauch. All mice were used at 8–12 wk of age, and all experiments used age- and sex-matched controls. Animals were housed in specific pathogen–free conditions at the Cornell East Campus Research Facilities and/or the Baker Institute for Animal Health Research facilities, the University of Washington South Lake Union 3.1 animal facility, or the Oregon Health and Science University Division of Comparative Medicine facilities. WT and *Gpr44^−/−^* female mice were littermates or were cohoused for at least 2 wk before experimentation (no differences were observed between cohoused nonlittermates and littermates in experiments performed). WT and *Gpr44^−/−^* mice were positive for *Tritrichomonas* species in the intestine. All experiments using organoids or analyses of IECs used littermates. All experiments were performed under protocols approved by the Cornell University, University of Washington, or Oregon Health and Science University institutional animal care and use committees.

### Type 2 intestinal inflammation and EdU treatment

The *N. brasiliensis* life cycle was maintained as previously described (Camberis et al., 2003). Mice were infected with 500 L3 *N. brasiliensis* larvae subcutaneously, and analyses were performed at day 7 p.i. unless specified otherwise. Adult worms were quantified directly from intestinal tissues as described previously (Camberis et al., 2003) on day 5 and day 7 p.i. For assessment of Type 2 intestinal inflammation, the MLNs were harvested. Single-cell suspensions of murine MLNs were prepared by mashing through a 70-µm cell strainer and counting total cells. To measure intestinal epithelial proliferation and distance traveled of labeled cells, mice were injected i.p. with 1 mg EdU in PBS on day 4 p.i., 24 h before euthanasia at day 5 p.i.

### Inflammasome-induced inflammation

*Bacillus anthracis* protective antigen (PA) was purified from *Escherichia coli* or insect cells as described previously (Rauch et al., 2017; von Moltke et al., 2012). Lethal factor (LF) N-terminus fused to *Vibrio parahaemolyticus* flagellin (LFn-VP FlaA) was cloned using *V. parahaemolyticus* serotype O3:K6 (strain RIMD 2210633) FlaA into pAcSG2 with a 6xHIS tag and purified as in Mitchell et al. (2020). Mice were injected with 0.8 µg/g body weight PA and 0.2 µg/g body weight LFn-VP FlaA retro-orbitally; 20 min after injection, animals were euthanized, and 2-cm pieces of ileum or jejunum were immediately flash frozen for eicosanoid analysis. To expand tuft cells and induce Cre expression, Pou2f3-CreERT2 *iNlrc4^+^* and Pou2f3-CreERT2 *Nlrc4^−/−^* littermates were treated for 7 d with 150 mM sodium succinate hexahydrate (Alfa Aesar) in the drinking water and tamoxifen chow (Envigo) ad libitum.

### Prostaglandin analysis

For prostaglandin analysis, 200–400 mg of small intestinal tissue was excised from naive and *N. brasiliensis*–infected mice at different time points. Tissues were snapped frozen on dry ice and stored at −80°C before being sent for analysis. Analysis was performed at the University of California, San Diego Lipidomics Core as previously described (Quehenberger et al., 2010) or at the University of California, Berkeley as follows: analytical synthetic standards for lipid mediators and polyunsaturated fatty acids were purchased from Cayman Chemical. Deuterated internal standards (PGE_2_-d4, LTB_4_-d4, 15-HETE-d8, LXA_4_-d5, DHA-d5, and AA-d8) were added to all samples before processing to calculate class-specific recoveries. Frozen tissues were placed in MeOH and processed in a refrigerated beat homogenizer. Supernatants were extracted using C18 solid-phase columns. Extracted lipids were analyzed by liquid chromatography–tandem mass spectrometry–based lipidomics using an AB SCIEX 4500 QTRAP mass spectrometer (Livne-Bar et al., 2017; von Moltke et al., 2012; Wei et al., 2020). Analysis was performed in negative ion mode using scheduled multiple reaction monitoring with one specific diagnostic transition ion for quantification and two to four transition ions for confirmation of each analyte. For quantification, calibration curves and HPLC retention times for each analyte were established with synthetic standards.

### Flow cytometry, fluorescence-activated cell sorting, and single-cell RNA transcript staining

Single-cell suspensions were incubated with Aqua Live/Dead Fixable Dye (Life Technologies) and fluorochrome-conjugated mAbs against mouse CD3 (17A2), CD4 (GK1.5), CD5 (53–7.3), CD11b (M1/70), CD11c (N418), CD19 (eBio1D3), CD25 (PC61.5), CD45 (30-F11), CD45.1 (A20), CD45.2 (104), CD127 (eBioSB/199), CD90.1 (HIS51), CD90.2 (53–2.1), IL-33R (RMST2-2), IL-25R (MUNC33), Klrg1 (2F1), NK1.1 (PK136), or Siglec F (E50-2440; BD Biosciences). All antibodies were from Thermo Fisher Scientific unless otherwise noted. For RNA staining analyses, cells were treated according to manufacturer’s instructions using a commercially available kit (PrimeFlow RNA Assay; Thermo Fisher Scientific) and compatible commercially available probes for *β2m* (used as a positive control for RNA staining) and *Gpr44* (Thermo Fisher Scientific). Eosinophils and ILC2s were gated as live, CD45^+^Siglec F^+^CD11b^+^ and live, CD45^+^lin^−^CD127^+^Klrg1^+^IL-25RB^+^Gata3^+^, or live, CD45^+^lin^−^CD127^+^CD25^+^ST2^+^CD4^−^, respectively. IECs for flow RNA staining were gated as live, CD45^−^*β2m*^+^EpCAM^+^.

For primary epithelial cell staining and sorting, stripped IECs were prepared, and for intestinal organoid epithelial cell staining and sorting, IECs were harvested from intestinal organoids by dissociating the organoids in TrypLE Express Enzyme (1×), no phenol red (Sigma-Aldrich), followed by washing and staining in flow cytometry buffer. IECs were stained in 100-µl volume of flow cytometry buffer with anti-mouse CD166 Fitc (eBioALC48), anti-mouse EpCAM PE (G8.8), anti-mouse CD24 PerCP Cy5.5 (M1/69), anti-mouse CD45 eFluor 450 (30-F11), anti-mouse Siglec F PE-Texas Red (E50-2440; BD Biosciences), biotinylated Ulex Europaeus Agglutinin 1 (UEA 1; Vector Laboratories), and Aqua Live/Dead Fixable Dye (all from Thermo Fisher Scientific unless otherwise noted) for 30 min. Cells were then washed twice in flow cytometry buffer and incubated for 15 min in streptavidin APC (Thermo Fisher Scientific). Fully stained cells were filtered through a 40-µM filter and sorted using a four-laser FACSAria II (BD Biosciences) with a 100-µm nozzle at 20 psi. Goblet cells, tuft cells, and enterocytes were defined as live, CD45^−^EpCAM^+^UEA 1^+^CD24^−^CD166^−^ (Knoop et al., 2015; Nefzger et al., 2016; Smith et al., 2017; Wong et al., 2012), live, CD45^−^EpCAM^+^Siglec F^+^CD24^+^, and live, CD45^−^EpCAM^+^UEA 1^−^ CD24^−^CD166^−^, respectively. Other epithelial cells, stem cells, and Paneth cells were sorted from previously characterized reporter mice systems. ISCs from Lgr5-GFP mice and Paneth cells from the Defa2-CreERT:Rosa26-tdTomato mice were sorted and kindly provided by the Dekaney laboratory at North Carolina State University (King et al., 2013). All samples were run on a four-laser LSR II (BD Biosciences) or a Gallios (Beckman Coulter) flow cytometer, and FlowJo 10 (Tree Star, Inc.) was used to analyze data.

### Preparation and isolation of epithelial and immune cells from skin and lung tissue

Dorsal skin samples collected from WT C57BL/6 mice were digested in 1 U/ml Liberase TL and 100 μg/ml DNase for 90 min. A single-cell suspension was generated by mashing skin digest through a 40-µM nylon filter. For lungs, the parenchyma was perfused with PBS and then inflated with 1 U/ml of Dispase and 200 μg/ml DNase and incubated at 37°C for 45 min, then digested with Liberase TL (per manufacturer's instructions) and 200 μg/ml DNase. Lung digest was poured through a 40-µM filter for epithelial cells, and the rest of the tissue was mashed for immune cell isolation. The single-cell suspensions were treated with RBC ACK lysis buffer. CD45^−^ and CD45^+^ cells were enriched from single-cell suspensions using the CD45 positive-selection MACS (Miltenyi) kit.

### scRNA-seq

scRNA-seq was performed using the 10× Genomics Chromium instrument. Initial quality assessment and alignment to the mouse genome was completed using 10× Genomics Cellranger software. Analysis was done using Seurat (3.0.1). Cells with <750 genes/cell, >15,000 reads/cell, or >15% of genes being mitochondrial were removed, resulting in a total of 2,651 and 1,892 cells for WT and *Gpr44^−/−^* groups, respectively. scRNA-seq data are available under Gene Expression Omnibus accession no. GSE148694 or were accessed from publicly available datasets.

### Low-input bulk RNA-seq

IECs from organoids prepared as indicated below (400 cells) were sorted directly into lysis buffer from the SMART-Seq v4 Ultra Low Input RNA Kit for Sequencing (Takara), and reverse transcription was performed followed by PCR amplification to generate full-length amplified cDNA. Sequencing libraries were constructed using the NexteraXT DNA sample preparation kit with unique dual indexes (Illumina) to generate Illumina-compatible barcoded libraries. Libraries were pooled and quantified using a Qubit Fluorometer (Life Technologies). Sequencing of pooled libraries was performed on a NextSeq 2000 sequencer (Illumina) with paired-end 59-base reads, using NextSeq P3 sequencing kits (Illumina) with a target depth of 5 million reads/sample. Base calls were processed to FASTQs on BaseSpace (Illumina), and a base call quality-trimming step was applied to remove low-confidence base calls from the ends of reads. The FASTQs were aligned to the GRCm38 mouse reference genome using STAR v.2.4.2a, and gene counts were generated using htseq-count. Quality control metrics were calculated using the Picard family of tools (v1.134).

All libraries met quality thresholds of at least 2.5 × 10^6^ total reads, at least 80% of reads aligned to the genome, and median coefficient of variation of coverage <1. RNA-seq count data were filtered to protein-coding genes with at least one count/million in 10% of the libraries and were normalized using the trimmed mean of M values (Robinson and Oshlack, 2010). Differential gene expression analysis was conducted with limma (Ritchie et al., 2015), using contrasts extracted from a single model containing all conditions. P values were adjusted for multiple testing (Benjamini and Hochberg, 1995), with a threshold of adjusted P < 0.05 to identify differentially expressed genes. Heatmaps were generated with Complex Heatmap (Gu et al., 2016).

Gene lists were generated for organoid genes up-regulated or down-regulated by IL-4+IL-13 treatment compared with media, whose expression status was reversed by PGD_2_ treatment via CRTH2. Functional analysis of these two gene lists was done with IPA (Qiagen; https://www.qiagen.com/ingenuity). IPA utilizes a right-tailed Fisher’s exact test to determine if pathways are significantly altered between conditions. Functional pathways identified by IPA were then filtered on activation z-score >2.0 or z-score less than −2.0 for IL-4+IL-13 up-regulated and down-regulated genes, respectively, and the top 20 pathways identified with the highest −log10(P value) were plotted. For visualization of functional pathways, g:Profiler-identified biological process gene ontology terms associated with the DEG lists were clustered in Cytoscape v3.8 using EnrichmentMap, and related pathways were highlighted using the AutoAnnotate app (Reimand et al., 2019). All up-regulated genes were included in pathway analysis. For genes down-regulated by IL-4+IL-13, gene sets were filtered on log(fold change) less than −2.0.

### RNA extraction, cDNA synthesis, and real-time PCR from organoids and sorted cells

Sort-purified cells were collected in Buffer RL (Norgen Biotek). Total RNA was subsequently extracted and purified from these collected samples with use of a Single Cell RNA Purification Kit (Norgen Biotek) according to the manufacturer’s protocol. For small IEC–type marker analysis and assessment of *Gpr44* expression, cDNA was generated using the High Capacity RNA-to-cDNA kit (Applied Biosystems). Real-time PCR was performed with TaqMan Gene Expression Master Mix (Applied Biosystems) on equal volumes of each cDNA sample in triplicate on a Bio-Rad CFX96 Touch Real-Time PCR Detection System (Bio-Rad Laboratories) using the following TaqMan probes (Thermo Fisher Scientific): *Chga* (Cat# 4331182, Mm00514341_m1), *Dclk1* (Cat# 4331182, Mm00444950_m1), *Lgr5* (Cat# 4331182, Mm00438890_m1), *Lyz1* (Cat# 4331182, Mm00657323_m1), *Muc2* (Cat# 4331182, Mm01276696_m1), *Gpr44* (Cat# 4351372, Mm01223055_m1), *Rps9* (Cat# 4331182, Mm00850060_s1), and *Sis* (Cat# 4331182, Mm01210305_m1). *Rps9* was used as the housekeeping gene for normalization.

For human studies, expression data were extracted from mRNA harvested from biopsies obtained during the colonoscopies of healthy donors (*n* = 13, ages 24–72) or patients with Crohn’s disease (*n* = 9, ages 26–58) or ulcerative colitis (*n* = 10, ages 24–67) participating in a biorepository program. Biopsies were obtained from the ascending colon in healthy controls and from inflamed or uninflamed segments of colon in patients with inflammatory bowel disease. The latter were on prednisone (12%), 5′aminosalicylates (38%), 6-mercaptopurine (9%), and/or anti-TNF biologics (21%) or no medication for their condition (32%) at the time of sampling. Samples were placed in cold RNAlater (Thermo Fisher Scientific) immediately ex vivo and frozen at −70°C within 24 h. All subjects consented to participate in research according to a protocol approved by the Institutional Review Board of Virginia Mason Medical Center in accordance with the Declaration of Helsinki.

Intestinal organoids were harvested by dissolving Matrigel in Gentle Dissociation Reagent and/or cold PBS with RNAsin Plus RNase Inhibitor, 10,000 U (Promega) and washing with cold PBS; RNA was then isolated using the mirVana microRNA isolation kit (Thermo Fisher Scientific) or with the Single Cell RNA Purification Kit (Norgen Biotek) according to the manufacturer’s instructions. Real-time PCR was performed on cDNA generated using a Superscript II reverse transcription kit (Thermo Fisher Scientific) using SYBR green master mix (Applied Biosystems) and commercially available Quantitect (Qiagen) or Taqman (Thermo Fisher Scientific) primer sets. Samples were run on the ABI 7500 real-time PCR system (Life Technologies).

### BM chimeras and cell transfers

Single-cell suspensions from the BM of 8-wk-old mice were prepared by flushing the femur and tibia with DMEM (Corning), passing the cells through a 70-µm filter, and lysing RBCs with ACK buffer (Lonza). Recipients were lethally irradiated with 1,000 CyG and given 4 × 10^6^ donor cells i.v. Sulfamexosone/trimethoprim (Hi-Tech Pharmacal) was provided in the drinking water for 2 wk after reconstitution, and mice were analyzed at 6 wk after reconstitution.

### Crypt isolation and culture of murine organoids and intestinal monolayers

The crypts were isolated from the small intestine of mice according to the manufacturer’s instructions (Stem Cell Technologies). The culture was passaged by dissociating the organoids in Gentle Dissociation Reagent (Stem Cell Technologies), washing in Advanced DMEM/F12 Reduced Serum Medium (Thermo Fisher Scientific), and plating in 50 µl of fresh ice-cold Matrigel Growth Factor Reduced Basement Membrane Matrix (Corning) on Costar 24 multiple-well plates (Corning). Organoids were grown at 37°C with 5% CO_2_, and medium was replaced every 3 d according to the manufacturer’s instructions. Organoids were used for experimental purposes after three passages unless otherwise noted. Fully differentiated and matured organoids were stimulated with or without 250 ng/ml rmIL-4 and 250 ng/ml rmIL-13 (R&D Systems), 20 µM PGD_2_ (Cayman Chemical), 1 ng/ml rmIFN-γ (R&D Systems), and 1 µM CRTH2 inhibitor OC000459 (Cayman Chemical) between day 2 and day 6 after passage. PGD_2_ and OC000459 were reconstituted in DMSO, and DMSO was added in equivalent amounts to all conditions as vehicle control.

For IEC monolayers, intact epithelial crypts were isolated from the small intestine following the protocol described for organoid culture. After crypts were isolated, intestinal monolayers were made as previously described (McGinty et al., 2020). Crypts were resuspended in complete organoid media supplemented with 10 mM Y27632 (StemCell Technologies) and 10 mM SB431542 (StemCell Technologies) and then plated in warm plates precoated with Matrigel. Plates were coated with Matrigel by adding 100 µl of 2% Matrigel in cold DMEM to each well in 48-well plates and incubating at least 30 min at 37°C. After plating, cells were incubated overnight at 37°C to allow adherence, and medium containing unattached cells was replaced the next day, followed by an additional hour of incubation. Medium was then aspirated, and stimuli were added in HBSS with Ca^2+^ and Mg^2+^. The cells were incubated for 30 min at 37°C. Supernatants were collected and used to perform lipidomics as described above.

### Quantification of ISC organoid-forming efficiency and budding

Crypts isolated from WT and *Gpr44^−/−^* mice were suspended in Matrigel at a seeding density of 200 organoids/well. After 4 d in culture, the whole gel was imaged in brightfield using a ZEISS Axiocam 503 mono camera, and the number of buds/organoid was counted on days 4, 5, 7, and 10 after seeding. For organoid-forming efficiency, the number of growing organoids was quantified at day 7 after seeding, and the percentage of organoids formed/well was calculated.

### ELISA and real-time PCR

Cytokine levels in cell-free supernatants and tissue homogenates were assessed using standard sandwich ELISA for IL-4 and IL-13 (Thermo Fisher Scientific). For real-time PCR from tissues, RNA was isolated from intestinal tissue using TRIZOL Reagent (Thermo Fisher Scientific), according to the manufacturer’s protocol. Real-time PCR was performed on cDNA generated using a Superscript II reverse transcription kit (Thermo Fisher Scientific) using SYBR green master mix (Applied Biosystems) or Taqman Gene Expression Mastermix (Thermo Fisher Scientific) and commercially available Quantitect (Qiagen) or Taqman (Thermo Fisher Scientific) primer sets. Samples were run on the ABI 7500 real-time PCR system (Life Technologies).

### Histology and immunofluorescence

At necropsy, 1-cm length of the intestine was excised from a region ∼10 cm into the small intestine (jejunum) and fixed in 4% (vol/vol) paraformaldehyde until tissue embedding. Tissues were then paraffin embedded, and 5-µm sections were stained with PAS/Alcian blue. Image acquisition was performed using a ZEISS AxioObserver Z1 Carl ZEISS Axio Camera and ZEISS ZEN Microscope acquisition software. Adobe Photoshop was used to adjust brightness, contrast, and color balance (changes were applied to the whole image) in the same way to all representative images. For quantitative measurement of goblet cells, a blinded counting of the number of PAS/Alcian blue–positive cells in the transverse section of an average of 50 villi was performed.

For Dclk1 or Klrg1 staining, paraffin-embedded sections were deparaffinized and rehydrated. Antigen retrieval was performed using sodium citrate buffer. Sections were permeabilized with 0.1% Triton-X in PBS (Sigma-Aldrich) and blocked in 5% goat serum or 5% mouse serum, respectively, in Tris/NaCl blocking buffer (TNB; 0.1 M Tris-HCL, 0.15 M NaCl, and 5 mg/ml TSA blocking reagents [Perkin Elmer], pH 7.5). Following blocking, sections were incubated with primary antibody (anti-Dclk1, clone ab31704; Abcam or anti-Klrg1 Alexa Fluor 488; clone 2F1; Thermo Fisher Scientific) overnight at 4°C or for 1–2 h at ambient temperature in 5% goat or mouse serum in TNB. For Dclk1, following washing with PBS, sections were incubated with the secondary antibody (anti-rabbit IgG F(ab′)2-AF594) for 1 h in TNB blocking buffer. Image acquisition was performed using a ZEISS Axiocam 503 mono camera. Tuft or Klrg1^+^ cell number was calculated using ImageJ software to manually quantify Dclk1^+^ or Klrg1^+^ cells/crypt/villus unit in each section, counting at least 15 and up to 45 units/section where possible for Dclk1 and at least 10 and up to 15 units/section where possible for Klrg1, with measurements averaged to generate a single value for each animal.

For EdU staining, paraffin-embedded sections were deparaffinized and rehydrated and then stained for EdU using the Click-iT Edu Imaging Kit (Thermo Fisher Scientific) according to manufacturer’s instructions. Slides were mounted with Vectashield with DAPI (Vector Laboratories) and imaged with a Nikon A1R Ti Confocal microscope with an Eclipse inverted microscope using the 20× objective lens with a 0.75 numerical aperture. Images were acquired with Nikon Elements acquisition software. EdU signal in individual crypt/villus units was calculated using ImageJ software by outlining the crypt/villus unit to be measured and then measuring the mean fluorescence intensity (MFI) of EdU and DAPI to generate a ratio of EdU:DAPI, to normalize EdU staining to total DNA staining (DAPI). For each mouse, at least 10 crypt/villus units were measured and then averaged to generate a single value for each animal. To measure the EdU distance traveled, the length from crypt base to villus tip of individual crypt/villus units was measured in ImageJ, and then the distance that EdU^+^ cells had traversed was measured from the base of the crypt to the leading edge of EdU staining (measuring on one side of the crypt/villus unit only, selecting the side where the EdU had moved farthest). For each mouse, at least 10 crypt/villus units were measured and then averaged to generate a single value for each animal.

### Organoid immunofluorescence staining

Organoids were grown in Matrigel on 8-well chamber slides with removable wells (Thermo Fisher Scientific) and fixed after exposure to specific treatments in PBS containing 4% paraformaldehyde (pH 7.4) and 2% sucrose for 20–30 min at room temperature. This was followed by permeabilization in 0.2% Triton X-100 for 30 min at room temperature and staining for EdU using the Click-iT EdU Imaging Kit (Thermo Fisher Scientific) before proceeding to antibody staining (if applicable). For EdU staining, free aldehydes were blocked with 100 mM glycine for 1 h at room temperature, followed by blocking of nonspecific antibody binding using 2% normal goat serum and 1% BSA. Primary antibody against Muc2 (clone H-300; Santa Cruz Biotechnology) was diluted in immunofluorescence buffer (1% normal goat serum and 0.5% BSA), and staining was done overnight at 4°C or for 1 h at room temperature with slow agitation. This was followed by staining with goat anti-rabbit Alexa Fluor 594 (Thermo Fisher Scientific), incubating in immunofluorescence buffer overnight at 4°C or for 1 h at room temperature. Nuclei were stained with DAPI (Sigma-Aldrich) in water at a final concentration of 1 µg/ml. Organoids were mounted using Fluoromount-G (Thermo Fisher Scientific) and imaged with the Nikon A1R Ti Confocal microscope with an Eclipse inverted microscope using the 20× objective lens with a 0.75 numerical aperture. Images were acquired with Nikon Elements acquisition software and processed and analyzed using Image J software. For each condition or genotype in each experiment, 5–10 (condition) or 15–20 (genotype) individual organoids were assessed, and the EdU or Muc2 signal in individual organoids was calculated using ImageJ software by outlining the organoid and then measuring the MFI of EdU or Muc2 and DAPI to generate a ratio of EdU:DAPI or to quantify the MFI of Muc2.

### Statistics

Results in graphs are displayed as mean ± SEM using Prism version 7 (GraphPad Software, Inc.) except where mentioned. Statistical outliers were identified in normal Gaussian datasets using the extreme studentized deviate method, and outliers were uniformly omitted. Statistical analysis was performed using JMP software (SAS). Unless indicated otherwise, data were analyzed using linear mixed-effects models with a fixed effect of experimental group and a random effect of experiment day. Model assumptions of normality and homogeneous variance were assessed by a visual analysis of the raw data and the model residuals. Right-skewed data were log or square root transformed. Experimental group was considered statistically significant if the fixed effect F test P value was ≤0.05. Post hoc pairwise comparisons between experimental groups were made using Tukey’s honestly significant difference multiple-comparison test. A difference between experimental groups was taken to be significant if the P value (Prob > F) was less than or equal to 0.05 (*, P ≤ 0.05; **, P ≤ 0.01; ***, P ≤ 0.001). We performed power analyses (power > 0.8 and α = 0.05) on preliminary datasets to determine the number of mice required for each readout (in total across all experiments). For bioactive lipid prostaglandin analytes measured in a time course of infection, Dunnett’s test was done to identify difference between control time point (day 0) and every other time point examined (prostaglandin data from *Pou2f3^−/−^* mice were analyzed as above using JMP, and data from *Stat6^−/−^* mice were analyzed using a one-way ANOVA in JMP). For human data and prostaglandin data in monolayers, Vil1-Cre*^+^* i*Nlrc4^+^* and controls, or Pou2f3-CreERT2*^+^* i*Nlrc4^+^* mice and controls, statistical analysis was performed using GraphPad Software. Data were analyzed using the unpaired Student’s *t* test or a parametric one- or two-way ANOVA. Relative gene expression data for *Gpr44* or other lineage marking genes in various cell types were not subjected to statistical analysis. Since analyses were performed with a control sample set as the normalizer, statistical analysis comparing against that control value does not impute biological significance. For example, detectable relative expression of *Gpr44* in a given cell type that happens not to be statistically different from the positive control does not mean that expression in that given cell type is not biologically significant. Thus, we describe relative expression as “enriched” over the control.

### Online supplemental material

Fig. S1 shows gating strategies for MLN ILC2s, eosinophils, and Th2 cells and quantification of MLN ST2^+^ ILC2s by flow cytometry, Klrg1^+^ cell numbers by immunofluorescence staining, and IL-4 and IL-13 levels by ELISA in the intestine in WT and *Gpr44^−/−^* mice following *N. brasiliensis* infection. Fig. S2 shows quantification of MLN ST2^+^ ILC2s and eosinophils by flow cytometry and Dclk1^+^ cell numbers by immunofluorescence staining in WT and *Gpr44^−/−^* chimeric mice following *N. brasiliensis* infection. Fig. S3 shows *Gpr44* expression in skin and lung epithelial and immune cells and enrichment of IEC lineage-associated genes in sorted IEC cell types. Fig. S4 shows prostanoid levels in the small intestine of WT mice and PGD_2_ levels in the small intestine of WT and *Stat6^−/−^* mice following *N. brasiliensis* infection, a Uniform Manifold Approximation and Projection (UMAP) of WT IECs from naive mice, PGD_2_ levels in the small intestine of Vil-Cre iNlrc4^+^ or *Nlrc4^−/−^* mice treated with PBS or FlaTox, controls for IEC staining, and frequencies of tuft cells in WT and *Gpr44^−/−^* organoids. Fig. S5 shows *Lgr5* expression in WT and *Gpr44^−/−^* mice following *N. brasiliensis* infection and *Lgr5* expression, TUNEL staining, expression of goblet- and tuft-associated genes, and Cytoscape visualization of pathway analysis from RNA-seq data in organoids treated with Type 2 cytokines with or without PGD_2_ and/or a CRTH2 inhibitor.
